# Passive Ice Protection Systems for Unmanned Aerial Vehicles Applications: A Review

**DOI:** 10.1002/smll.202412465

**Published:** 2025-04-21

**Authors:** Lorenzo Facco, Riccardo Parin, Maria Basso, Alessandro Martucci, Elena Colusso

**Affiliations:** ^1^ Department of Industrial Engineering University of Padova and INSTM Via Marzolo 9 Padova 35131 Italy; ^2^ Terra X Cube Eurac Research Via Ipazia 2 Bolzano 39100 Italy

**Keywords:** anti‐icing, ice adhesion, icephobic coating, icephobic surface characterization, passive ice protection, superhydrophobic

## Abstract

Unmanned aerial vehicles (UAVs) are promising platforms for operations in alpine regions due to their compact size, advanced camera systems, and ability to take off and land in confined areas. In such conditions, one of the most significant challenges for UAVs is operating in icing environments, as ice accretion can compromise the aerodynamics of the propellers and can potentially lead fto a loss of control and vehicle failure. To date, active de‐icing solutions, such as electrothermal systems, have been employed in the aeronautical sector; however, these systems are energy‐intensive. This review addresses passive ice protection systems from a material science prospective, by focusing on coatings which mitigate ice formation without energy consumption. A comprehensive description of the strategies to design an icephobic surface is presented and the state‐of‐the‐art icephobic coatings are analyzed, such as superhydrophobic surfaces, elastomers, liquid infused surfaces, gels, polyelectrolytes, sol gel coatings, metal‐organic frameworks. A key focus is devoted to the characterizations for assessing ice mitigation of such coatings, i.e., contact angle and hysteresis measurements, and to the correlation between durability and number of icing and de‐icing cycles. The most relevant solutions for aerial vehicles are described in the final part of this review.

## Graphical abstract

This review summarizes the recent progress on passive ice protection systems for aeronautical applications, with a particular focus on drones. It provides a general strategy for designing and characterizing an icephobic surface and a state‐of‐the‐art account of icephobic coatings. The perspectives for future challenges in developing anti‐icing passive systems are addressed.

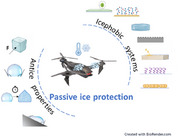

## 1 Introduction

In recent years, the field of UAVs has shown great technological progress and has been applied to several new applications, such as recognition in military operations, aerial photographs, delivery systems,^[^ ^1^ ^]^ and emergency rescue of people.^[^ ^2^, ^3^ ^]^ Particularly in alpine regions, drones might have a large range of applications. It has been reported that drones, owing to their GPS tracking system combined with a specific camera (optical, night vision, or infra‐red camera), can be adopted to rescue hikers or mountaineers, to map areas affected by a natural disaster, or to transport emergency packs.^[^ ^4^ ^]^ When operating in alpine environment, UAVs constitute a valid alternative to helicopters. On one hand, drones are advantaged by the small size of the vehicle, which can land and take off in valleys, woods, canyons, places in which helicopters could never reach.^[^ ^5^ ^]^ On the other hand, the adoption of UAVs is more cost‐effective than helicopters. In addition, the rescue of hikers and the transportation and supply of emergency kits by drones could be faster than the traditional use of helicopter or the terrestrial search of the people.^[^ ^4^ ^]^ The speed factor is a crucial parameter when operating in emergency situations, especially in mountains. As it is declared by the association “Wilderness Medical Society” in the protocol about prevention and management of avalanche and non‐avalanche snow burial accidents, the highest probability of a successful rescue operation is manifested when the injured mountaineer is assisted within 60 min after the accident.^[^ ^6^ ^]^

**Figure 1:**
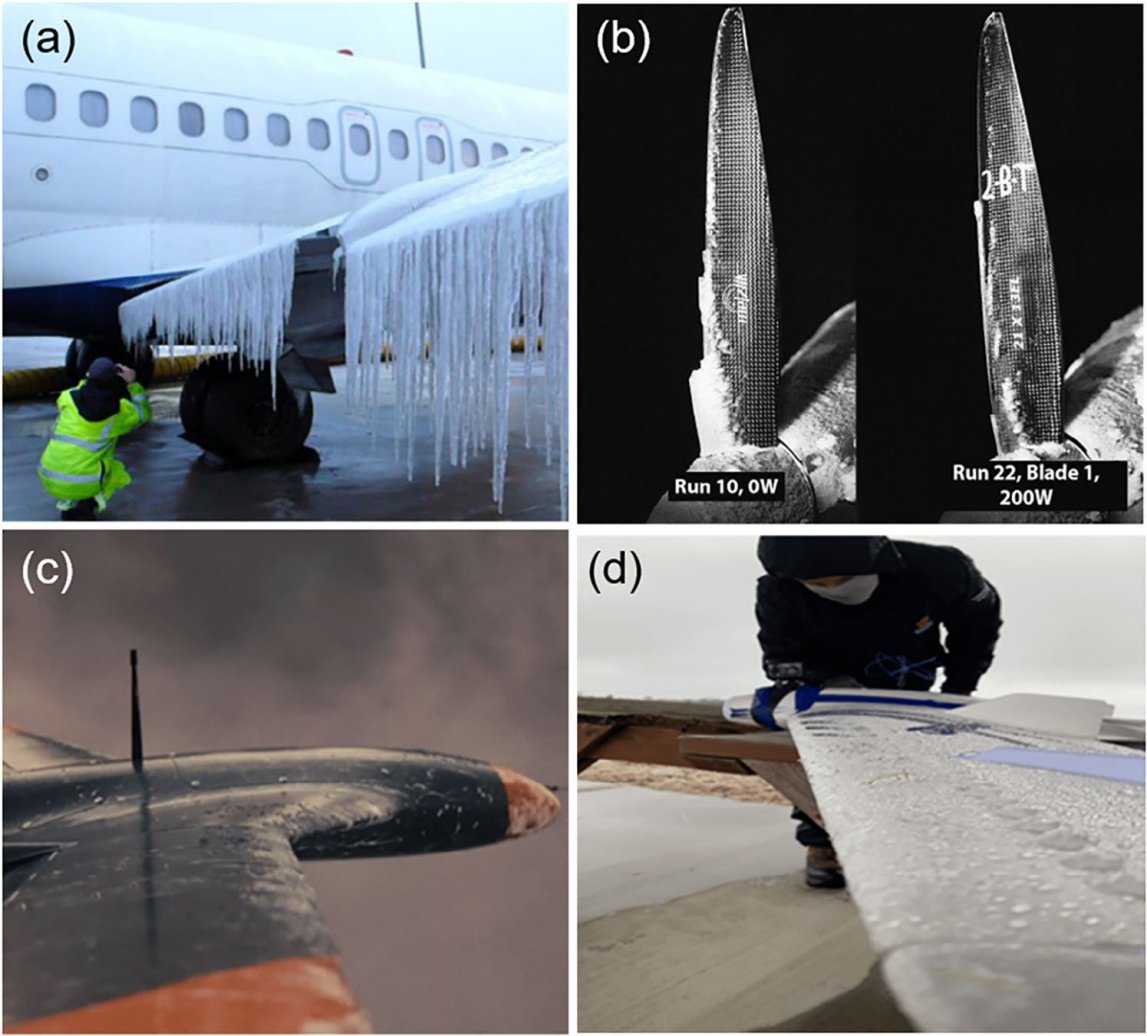
Ice accumulation on the wings of an airplane (a), on rotatory propeller drones (b) and on fixed wings drones (c,d). a) Adapted with permission.^[^ ^20^ ^]^ Copyright 2019, Elsevier. b) Reproduced with permission under terms of the CC‐BY license.^[^ ^21^ ^]^ Copyright 2023, Elsevier. c,d) Reproduced with permission.^[^ ^22^ ^]^ Copyright 2022, MDPI.

At high altitudes and low temperatures, the critical role drones play in monitoring these areas has scarcely been reported, primarily due to the lack of data under such extreme conditions.^[^ ^7^, ^8^ ^]^ A significant challenge in these environments is ice formation,^[^ ^9^ ^]^ which poses a substantial obstacle for most UAVs, especially small rotary‐wing models. In‐flight icing occurs when a flying vehicle enters a cloud of supercooled water droplets, which freeze upon impact, causing ice accretion. This poses a serious threat to rotary‐wing UAVs in ice‐prone regions, potentially leading to loss of control and catastrophic failure (**Figure** **1**). About 25% of US military UAV missions in North America encounter icing hazards.^[^ ^10^ ^]^ In Denmark, adverse weather limits rotary‐wing UAV operations to 54% of the year, significantly less than fixed‐wing counterparts.^[^ ^11^ ^]^ In northern Europe, icing conditions can occur 35% to 80% of the time, with potential icing throughout the entire year in Norway, according to the season.^[^ ^12^ ^]^ Ice accumulation primarily affects propeller blades, impairing aerodynamic performance and increasing the risk of vibrations and imbalance due to ice shedding.^[^ ^13^, ^14^ ^]^ In 2018, the European Union adopted a new Unmanned Aircraft Systems (UAS) regulatory framework, which is a proportional risk‐based approach to UAS. This rule identified three categories of operation: basic, specific, and certified.^[^ ^15^ ^]^ The category specific requires authorization by the competent authority that goes through the definition of the Specific Operation Risk Assessment (SORA), which defines the SAIL (Specific Assurance and Integrity Level). When SAIL is level IV or higher a specific Design Verification Process (DVP) is required.^[^ ^16^ ^]^ So, if a risk of flight in icing conditions is foreseen, it must be shown that the vehicle is either able to sustain these conditions safely (ice protection system IPS),^[^ ^16^, ^17^, ^18^ ^]^ or has means to detect and avoid or safely exit those conditions with the so‐called ice detection systems (IDSs).^[^ ^19^ ^]^

Ice protection systems are divided into two groups: active or passive protection systems.^[^ ^23^ ^]^ Hybrid active/passive systems that combine both active and passive methods are also adopted in ice protection.^[^ ^23^ ^]^ Active systems remove ice accumulation by providing energy. The most used strategies in active protection are the electrothermal method and the hot air flow. In the electrothermal method, electricity flows through an electrical resistance, generating heat that melts the ice due to the Joule effect.^[^ ^24^ ^]^ In the hot air flow method, warm air from the engine is used to constantly heat the wings.^[^ ^25^ ^]^

On the other hand, passive systems have been studied and developed nowadays. These systems protect against icing by exploiting the properties of surfaces and natural forces such as wind, gravity, centrifugal force, and vibrations, without needing to supply energy.^[^ ^26^ ^]^ The European Union, in line with the Green Deal initiatives, promotes the adoption of alternative protection systems, through the Passive Ice Protection Systems (PIPS) project. These systems are more environmentally friendly, less energy demanding, and produce lower CO_2_ emissions compared to the traditional thermal‐electro methods. One common passive solution in aircraft is the use of propylene glycol, which has a low melting point and can form liquid solutions at −60 °C. This method is typically applied to wide‐body aircraft.^[^ ^27^ ^]^ One of the drawbacks of this approach is the requirement of 35 gallons of propylene glycol to guarantee effective ice protection and 150 gallons to completely remove ice from the aircraft.^[^ ^27^ ^]^ Successful ice protection can be guaranteed by tuning the properties of the surface, as we will discuss in detail in Section 4. Indeed, surface properties can be modified directly through techniques like texturing^[^ ^28^, ^29^ ^]^ or by adding a coating layer with specific functional groups and key properties. Examples of surface treatments used in everyday applications are protective layers for corrosion prevention in metals,^[^ ^30^ ^]^ anti‐reflective layers for optoelectronic devices,^[^ ^31^ ^]^ coatings for self‐cleaning applications,^[^ ^32^ ^]^ and coating treatments to promote dropwise condensation.^[^ ^33^, ^34^, ^35^, ^36^, ^37^ ^]^ Recently, surface treatments designed for passive ice protection have been developed in response to current environmental research priorities.^[^ ^9^ ^]^ Nanoengineered coatings based on functional nanoparticles or polymers in liquid media can be prepared using methods such as dip coating,^[^ ^38^ ^]^ spin coating,^[^ ^39^ ^]^ or spray coating.^[^ ^40^ ^]^ Alternatively, the coating material can be deposited from a vapor phase, through physical^[^ ^41^ ^]^ or chemical vapor deposition.^[^ ^42^ ^]^

Hybrid systems combine the surface properties of a passive system with the energy demand of an active one (i.e., electrothermal or photothermal heating, electromechanical forces). For example, De Moral and coworkers have developed a hybrid system in which a piezoelectric material, LiNbO_3_, is coated with a layer of fluorinated zinc oxide (ZnO).^[^ ^43^ ^]^ In this system, the combination of the acoustic waves generated by the piezoelectric material when an electric field is applied to the system with the surface properties of the coating reduce ice adhesion and facilitate ice detachment.^[^ ^43^ ^]^ Recent research has increasingly focused on hybrid systems utilizing photothermal heating. In comparison to electrothermal heating techniques, photothermal systems demonstrate enhanced energy efficiency, as they harness solar energy as an external stimulus. These systems exploit materials with solar‐to‐heat conversion properties embedded in hydrophobic coatings, which enable surface temperatures to exceed the melting point of ice, thus promoting ice melting at the interface. Recent review papers^[^ ^44^, ^45^ ^]^ highlight the advantages of these hybrid systems, including improved reliability, efficiency, and energy‐saving anti‐icing capabilities. However, it is important to note that their efficiency may be limited in the cold, cloudy conditions typically encountered in mountainous regions, where emergency rescue UAVs are designed to operate.

This review defines and describes the key characteristics of an icephobic surface, explains the mechanism of ice formation, and discusses the most relevant strategies for delaying icing on a surface. Additionally, it examines the state of the art of the most common ice passive protection systems, with a particular focus on aeronautical applications.

## 2 Ice Formation Mechanism

A crucial aspect of defining an anti‐icing strategy is understanding the mechanism of ice crystallization and kinetics on a substrate. This is particularly relevant for passive coating layers, as their surface is designed to delay or prevent successful water crystallization. In the scenario of icing on UAV propellers, the process begins when the UAV enters icing conditions, typically characterized by clouds containing supercooled liquid droplets. In such clouds, droplets typically remain liquid down to temperatures around −20 °C, after which ice crystals begin to form and coexist with the supercooled droplets.^[^ ^46^ ^]^ These droplets deposit onto the substrate, forming an initial dispersed population of water droplets. This paragraph provides an essential overview of the key features of this process, including its driving force and main phases: ice nucleation and subsequent ice propagation across the surface.^[^ ^47^ ^]^ Ice formation occurs when liquid or vapor water transitions into solid ice due to heat transfer from the water droplet to the environment under supersaturation conditions.^[^ ^47^ ^]^

### 2.1 Nucleation

**Figure 2:**
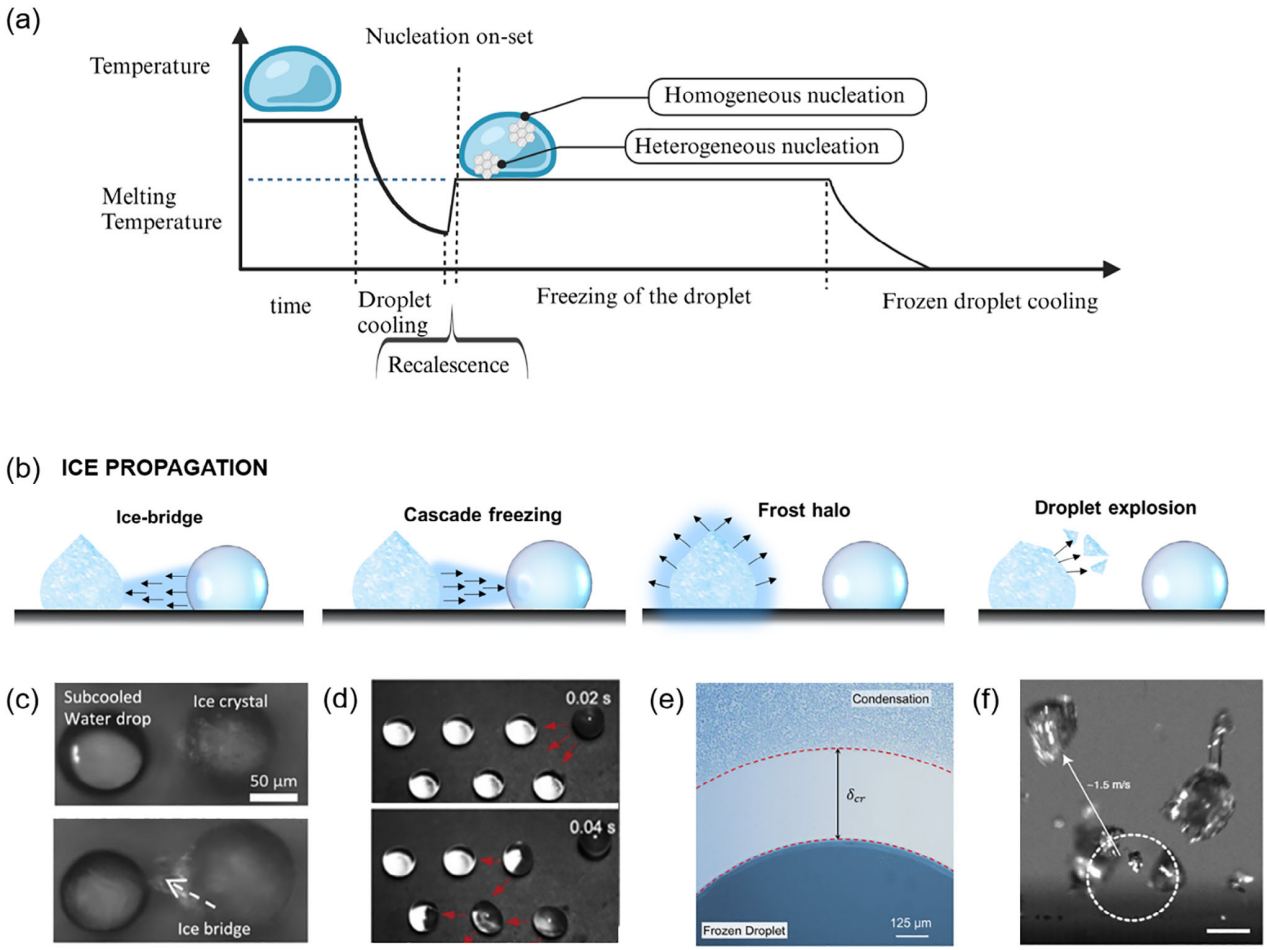
a) Timescale of icing showing the main stages: cooling of the droplet, recalescence (due to release of latent heat), and freezing. The temperature is constant from the formation of the very first ice nuclei until (nucleation on‐set) the complete freezing of the droplet. At the end of the phase transition, ice starts cooling. Nucleation may be homogeneous if it begins inside the droplet or heterogenous if the process starts at the surface/droplet interface. b) Schematic of ice propagation mechanism. Ice can propagate through: c) Ice bridges between an ice crystal and a supercooled water particle. Reproduced with permission.^[^ ^59^ ^]^ Copyright 2016, AIP Publishing. d) Cascade freezing due to the release of the latent heat from one frozen droplet to the supercooled droplets in the surroundings. Adapted with permission.^[^ ^60^ ^]^ Copyright 2018, American Chemical Society. e) Frost halo due to the freezing of condensate vapor. Reprinted with permission.^[^ ^61^ ^]^ Copyright 2016, American Chemical Society. f) Freezing of supercooled droplet due to ice shatters from an exploded ice crystal. Reprinted with permission.^[^ ^62^ ^]^ Copyright 2017, the American Physical Society.

Nucleation can occur in two forms: homogeneous, in which ice nuclei form due to random fluctuations in pure water, or heterogeneous, in which ice nuclei grow around pre‐existing particles or on solid interfaces.^[^ ^48^ ^]^ In the case of heterogeneous nucleation, the presence of an interface lowers the energy barrier, accelerating the process if compared to homogeneous nucleation. Heterogeneous nucleation is predominant in real‐world conditions due to the presence of atmospheric particles, such as dust, as well as surface irregularities and defects.^[^ ^47^ ^]^ Consider the simplified scenario of a droplet deposited onto a cooled substrate (**Figure** **2**a). In the first stage, the droplet temperature decreases due to heat transfer to the cold substrate until heterogeneous nucleation is initiated.^[^ ^47^ ^]^ Subsequently, recalescence occurs over approximately 10–100 ms, during which partial freezing releases latent heat. This heat release rapidly raises the droplet temperature to the freezing point, ultimately resulting in complete solidification.

This stage is slower and involves thermal exchange with the surroundings. Nucleation is typically seen as a stochastic event, where random oscillations caused by the Brownian motion causes the first nucleus to form.^[^ ^49^ ^]^ However, some studies suggest that specific active sites on surfaces enhance nucleation. These sites include defects such as cracks or pores and can be influenced by surface roughness and energy.^[^ ^50^, ^51^, ^52^ ^]^ Crystallographic parameters, such as the exposure of a specific crystallographic plane or the mismatch between surface and ice lattice parameters, affect the degree of ice nucleation. The chemical composition also plays a critical role in the effectiveness of an active site. The presence of a hydroxylated functional group can induce water to assume a hexagonal ice‐like structure and facilitates icing formation.^[^ ^53^ ^]^ According to these observations, ice nucleation can be considered as a single event rather than a stochastic process. Each active site is independent from the others and such transition may occur at a different temperature, due to variations in the Gibbs–Thompson effect or in capillary condensation. Evidence suggests that nucleation occurs close to an active site rather than randomly, and these sites remain consistent across thawing and de‐icing cycles.^[^ ^53^ ^]^

Icing events in aircraft are often further associated with impact icing. Impact icing is the freezing of a supercooled water droplet after the impact on a (cold) surface.^[^ ^54^ ^]^ When a water droplet hits a surface, it might spread on the surface (splash behavior) and then rebound.^[^ ^55^ ^]^ The energy from the impact can break the incoming droplet into small aggregates of water, which can then start a sequence of spreading and rebounding until the energy is fully dissipated. In terms of ice protection, the small droplets that result from the impact can be pinned to the surface, acting as ice nucleation sites for incoming subcooled water. The impact leads to a significant evaporation that drastically increases the supersaturation, causing water to solidify into ice.^[^ ^54^ ^]^ The behavior is strongly affected by the impact velocity, rebound height, surface morphology, and wetting properties. Since the impact velocity and rebound height depend on the vehicle's speed and environmental conditions, the only parameters that can be ad‐hoc designed are surface wettability and morphology.^[^ ^39^ ^]^

### 2.2 Ice Growth and Spreading

Crystal ice grow by attracting additional water molecules that freeze upon contact with the crystallization nuclei. When the first droplet freezes, a freezing wave spreads outward, solidifying the surrounding water molecules through several mechanism such as ice‐bridge, cascade freezing, and frost halo (Figure 2b).^[^ ^47^ ^]^

An ice bridge is one possible mechanism for ice propagation when the frozen process of the first droplet is finished. In this scenario, a frozen droplet is surrounded by supercooled liquid droplets. Because the vapor pressure of the solid droplet is lower than that of the supercooled one at the same temperature, a vapor flux from the supercooled droplet to the solid droplet let the formation of an ice bridge between them (Figure 2c). As soon as the ice bridge touches the liquid droplet, the latter immediately freezes and the same mechanism is repeated, leading to a domino effect.^[^ ^47^ ^]^

A transition from the ice bridge propagation mechanism to an alternative process may be observed when a second supercooled droplet undergoes freezing during the formation of the ice bridge. At this moment, the release of the latent heat of melting (recalescence) results in an increase in temperature and vapor pressure within the droplet. Consequently, the vapor may flow in the opposite direction, from the frozen droplet to the supercooled liquid, potentially leading to a cessation of ice bridge formation. The vapor emission creates a local supersaturation, which, in combination with airborne particles, facilitates the nucleation and freezing of the surrounding droplets upon contact. This process is reiterated every time a droplet freezes, resulting in a sequence of freezing cascade (Figure 2d). When the cascade freezing mechanism is dominating, the nucleation of the following droplets starts at the air/water interface instead of the surface/water interface.

A parallel freezing mechanism, which plays a significant role during the recalescence under low humidity conditions, is the frost halo (Figure 2e). In low humidity, evaporation rate of the first freezing droplet increases, reaching local supersaturation conditions. The evaporated water spreads radially from the initial droplet until the lower temperature causes the vapor to condensate and freeze. The phenomenon continues until the original droplet is completely frozen.^[^ ^47^ ^]^ The spatial extension of the frost halo depends on the equilibrium between the thermal conductivity of the surface and the relative humidity of the surrounding environment. The duration of this phenomenon is related to the heat exchange between the freezing droplet and the cold surface. If the heat diffusion from the cold surface to the droplet is low, the droplet will freeze slowly, resulting in a prolonged release of vapor.

In the last mechanism (Figure 2f), a water droplet begins to freeze from the outside. In absence of an airborne nucleating agent, the freezing process starts at the triple point and gradually covers the outmost layer of the droplet before progressing to the core. As the water freezes, it expands, leading to an increased pressure inside the droplet. This increased pressure results in the droplet's explosion, which activates ice‐shattering spreading. When ice fragments hit supercooled water, the liquid droplet begins to freeze. This represents the primary pathway for spreading when the freezing delay exceeds 1 s.^[^ ^47^ ^]^ Notably, this propagation mechanism is effective when the actual inter‐droplet distance is greater than 30 mm.

The wettability of a surface plays a crucial role in the ice growth and spreading. Liu et al.^[^ ^56^ ^]^ noted ice can growth in two main directions: it can spread along the surface with a dense population of ice crystals or grow off the surface, leaving empty spaces between two ice crystals. The along‐the‐surface growth is promoted by the presence of a bilayer of ice on hydrophilic surfaces (*θ* < 38°).^[^ ^56^ ^]^ This bilayer ice derives from the condensation and freezing of two layers of water molecules arranged in a hexagonal geometry on a hydrophilic surface.^[^ ^57^, ^58^ ^]^ The structural lattice alignment between the hexagonal bilayer and the forming bulk ice stimulates ice growth along the surface. In contrast, on more hydrophobic surfaces (*θ* > 40°), the ice bilayer is not present, and the out of surface growth is the preferred pathway.^[^ ^56^ ^]^

## 3 How to Characterize Icephobic Surfaces

**Figure 3:**
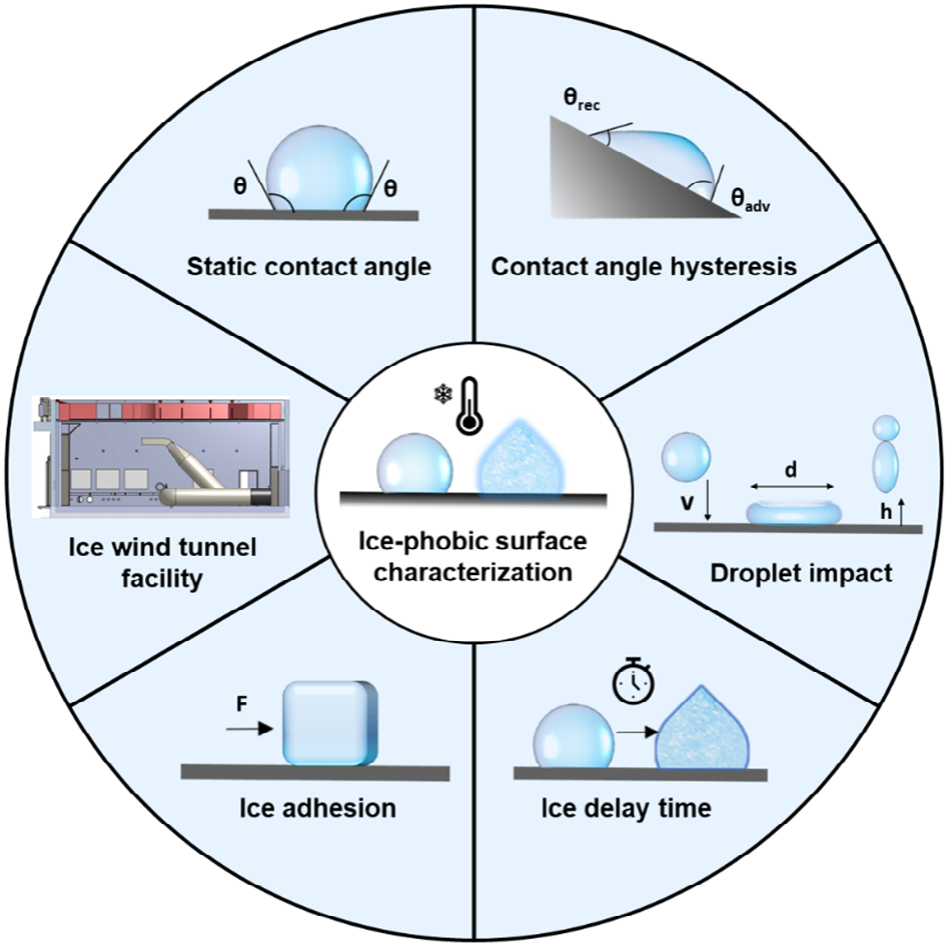
Summary of the main characterization techniques employed to evaluate the icephobic properties of a surface. The wettability of the surface can be characterized by CA, CAH, droplet impact measurements. The time ice accumulates on a surface (ice delay time) and its adhesion strength are other parameters evaluated. Real‐scenario experiments can be performed on ice tunnel facilities or climatic chambers to determine the performance of anti‐icing coatings over commercial devices.

An icephobic surface is defined as a surface that demonstrates the ability to either delay ice formation or reduce ice adhesion strength. Due to the absence of standardized criteria, all the designed surfaces predominantly target two specific aspects of ice formation: one category focuses on delaying ice nucleation,^[^ ^63^ ^]^ while another aims to reduce ice adhesion strength.^[^ ^64^ ^]^ According to the existing literature, only the surfaces which allow ice to be removed by shear forces below 100 kPa are considered as icephobic.^[^ ^9^, ^65^ ^]^ This benchmark aligns with the removal of ice accumulation by natural forces such as gravity, wind, or centrifugal forces. However, the above‐mentioned criterion is not sufficient to describe the complexity of the icing phenomenon. In this section, we will present a comprehensive series of tests that begin with the measurement of ice adhesion as illustrated in **Figure** **3**:

- Ice adhesion,^[^ ^64^, ^66^, ^67^ ^]^
- Static contact angle (CA),^[^ ^68^ ^]^
- Dynamic CA or sliding angle,^[^ ^64^, ^68^ ^]^
- Surface energy,^[^ ^68^ ^]^
- Drop impact,^[^ ^69^ ^]^
- Ice formation delay time icing/de‐icing experiments,^[^ ^70^ ^]^
- Ice tunnel experiments,^[^ ^71^ ^]^
- Cold chamber experiments^[^ ^7^ ^]^

There are several examples of experimental set‐ups for studying ice adhesion in literature. The most common method is the pushing rod method,^[^ ^67^ ^]^ where ice adhesion strength is evaluated by removing an ice block by a rod. Other typologies of tests, such as the centrifugal test,^[^ ^64^ ^]^ can also be found in various studies.^[^ ^72^ ^]^ Typically, these tests are conducted within an in‐house climate chamber that contains the test surfaces where ice is formed inside a mold.^[^ ^67^ ^]^

Ice adhesion is a macroscopic measure that quantifies the balance between adhesive forces (ice‐surface interactions) and cohesive forces (ice‐ice interactions). In the contexts of an ice block formed within a mold, the contribution of cohesive forces is influenced by several factors, including ice density, crystalline grains, and bond length. Adhesive forces primarily result from hydrogen bonding between the hydroxyl (−OH) groups present in ice and the polar groups on the surface. Electrostatic interactions have a minor effect on ice adhesion.

The variability in experimental outcomes can be attributed to the custom‐design of ice‐adhesion apparatuses,^[^ ^67^ ^]^ which includes factors such as rod shape, impact speed and impact height,^[^ ^73^ ^]^ freezing time, and type of ice used^[^ ^74^ ^]^ This diversity in setup may lead to discrepancy in results, complicating the comparison of findings across various studies reported in literature.

To compare different surfaces and the outcomes from different set‐ups, the value of shear force required to remove ice from a surface is normalized by the value of shear force needed to remove the same ice block from a reference aluminum surface. This ratio is called adhesion reduction factor.^[^ ^9^ ^]^

The second major aspect of icing is ice nucleation. Understanding ice nucleation of sessile droplets requires the characterization of surface wettability under icing conditions, as the nucleation energy barrier depends on wettability.^[^ ^47^, ^48^, ^63^, ^75^, ^76^ ^]^ The wettability of the surface describes the shape of water on a surface. A surface is considered (super)hydrophobic when a water droplet has a spherical shape, while it is hydrophilic when the droplet has a film‐like shape. When a droplet is on a flat surface (ideal case), three components should be considered to evaluate surface energy and droplet shape according to the Young's equation: the surface tension at the liquid/solid interface, the surface tension at the liquid‐air interface and the surface tension at the liquid–vapor interface.^[^ ^61^ ^]^ For a flat surface, the CA can vary from 0° to 120°. According to the value of Young's CA *θ*_Young_, a surface is classified as hydrophilic when the angle is 0° < *θ*_Young_ < 90°, while it is classified as hydrophobic when 90° < *θ*_Young_ < 120°.

However, a real surface is usually characterized by surface roughness, which influences the interaction with liquids. This roughness allows two possible wettability states: in the “Cassie–Baxter” state, air is trapped between the roughness and the droplet, while in the “Wenzel” state, the droplet wets the surface by filling the grooves, without any air present. Conventionally, the surfaces in which the Young angle is 120° < *θ*_Young_ < 180° are classified as superhydrophobic surfaces (SHSs).

Tuning the CA of a surface is not sufficient to achieve icephobicity, as this parameter does not adequately describe the dynamics of the droplets over the surface. Indeed, a key requirement for passive ice protection is the removal of water from the surface prior to freezing. To evaluate the efficiency with which a water droplet leaves the surface, conducting dynamic CA analysis is recommended. In this analysis, the contact angle hysteresis (CAH) can be evaluated as the difference between the average of the CA while the droplet is expanding (advancing contact angle, ACA) and the average of the CA while the droplet is contracting (receding contact angle, RCA). The smaller the CAH is, the faster the droplet leaves the surface. Alternatively, a droplet may be placed on a tilted plane. The angle the droplet starts rolling off is named sliding angle. The smaller the angle, the faster the water is removed from the surface. To measure these wettability properties, the CA goniometer is the selected instrument.^[^ ^77^ ^]^ The static CA is measured when a droplet of a specific volume is gently poured on a surface (sessile‐drop goniometry).^[^ ^68^ ^]^With the same experimental set‐up, consisting of a motorized syringe for precisely controlling the dispensed volume of water and a software‐operated camera for capturing images, ACA and RCA can be evaluated by increasing/decreasing the droplet volume.

**Figure 4:**
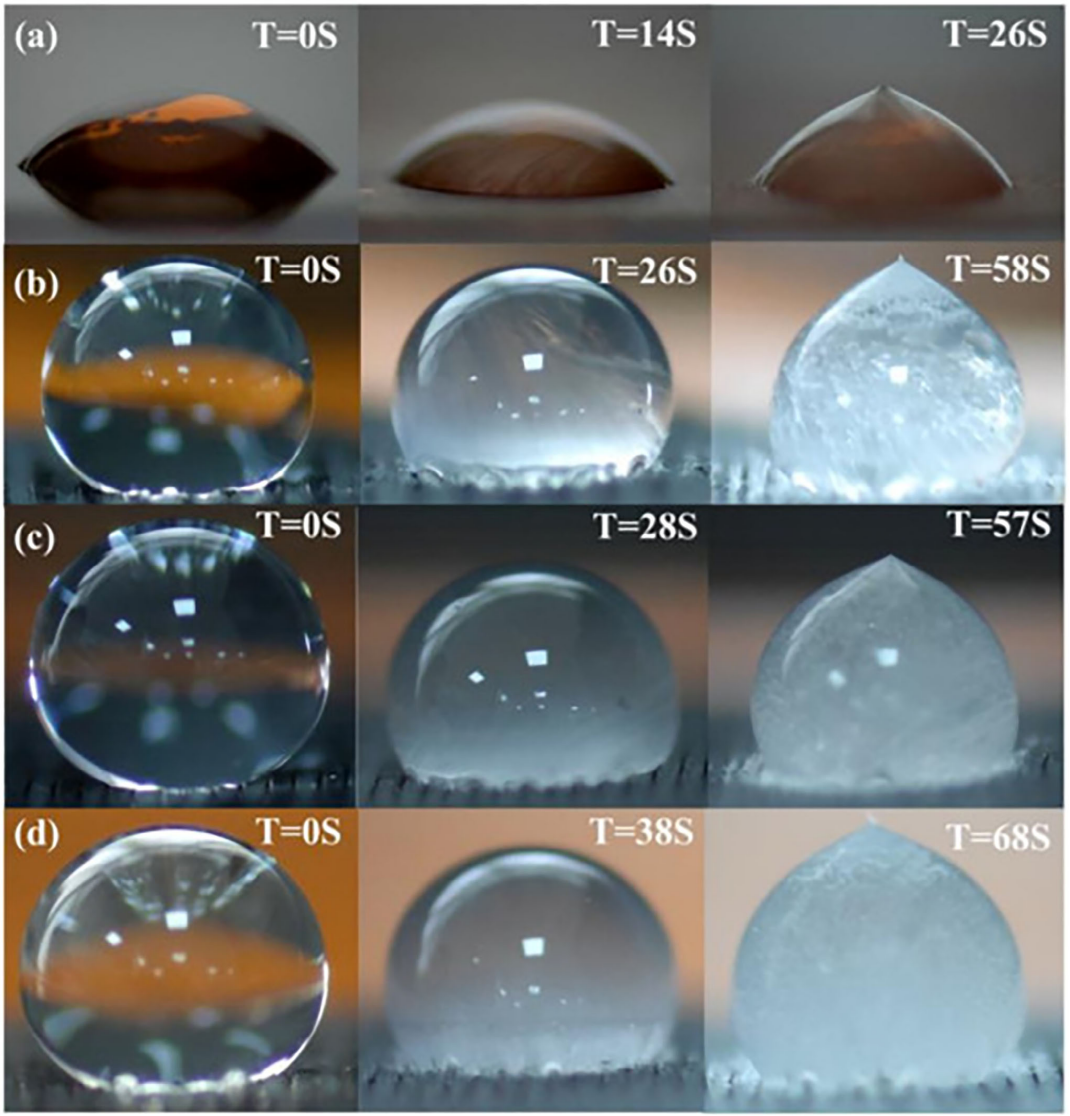
Digital images of delaying icing tests on microstructured graphene hydrophobic surfaces on polyimide film compared with an untreated one (a). The hydrophobic surfaces were prepared by laser direct writing employing different scanning speeds of 75 mm s^−1^ (b), 100 mm s^−1^ (c), and 125 mm s^−1^ (d). Reproduced with permission.^[^ ^78^ ^]^ Copyright 2023, MDPI.

A standard goniometer is not effective for icing purposes, due to the change of the surface energy in function of the decreasing temperature. An improvement of the experiment is generally achieved by the adoption of a cold module, i.e., a Peltier cell, as a sample holder. Additionally, the influence of the surrounding atmosphere should be considered, as the presence of condensable species, such as water vapor, can affect the experimental outcomes. To address this issue, it is suggested to conduct the experiment in a closed chamber purged with inert gas, such as nitrogen, to effectively remove the condensable species from the test environment. In this controlled set‐up, when water is poured on a substrate at a temperature below the melting point of water (for instance −10 °C), the duration required for a droplet to solidify is recorded and the results compared with those from a standard sample, for instance an untreated substrate, with the same experimental conditions (**Figure** **4**).

Drop impact experiments are performed in a closed chamber with inert atmosphere, similar to the CA measurements. In these experiments, a droplet is released from a fixed distance above the surface and impacts it at a certain speed. After the impact, the droplet may either bounce off completely, partially bounce or spread over the surface. The bigger the portion of water that bounce back, the better the anti‐icing performance of the coating.

The above‐mentioned tests are conducted on a laboratory scale. To effectively assess the potential real‐life applications of anti‐icing coatings, testing must also take place in specific cold chambers, where bigger and complex‐shaped objects are introduced (e.g., propellers, drones – Figure 3).

In cold chambers, environmental conditions typical of the high altitudes in mountains or other extreme cold environments can be simulated. These chambers allow for the evaluation of various forces such as gravity, centrifugal force, and wind. Specific tests can be conducted, including the ice wind tunnel (IWT) test or frozen rain test. During IWT tests, an airflow moves supercooled liquid droplets through the tunnel, impacting on the tested object. Typical parameters are liquid water content, droplet median volume diameter, speed of the air flow, and the temperature of the incoming cloud.^[^ ^79^, ^80^, ^81^ ^]^ In a frozen rain test, the effectiveness of anti‐icing systems is evaluated by exposing the tested object to simulated frozen water. This test aims to recreate environmental conditions that a drone might encounter while flying in extremely cold environments.

## 4 Strategies for an Effective Design of Surfaces for Passive Ice Protection

An icephobic surface is a surface designed to prevent or delay the freezing of condensed water on surfaces and to weaken the adhesion forces between ice and the surface itself.^[^ ^82^, ^83^ ^]^ To achieve these functions, several criteria must be satisfied:

1. The surface must have a low surface energy; thus, a SHS is the starting point for creating an icephobic surface^[^ ^84^ ^]^ by increasing the nucleation energy barrier.
2. The surface must suppress ice nucleation^[^ ^85^ ^]^ by depressing the freezing point.
3. The surfaces must have weak adhesion with ice,^[^ ^84^ ^]^ so it can be removed by natural forces such as wind, gravity, vibrations, and centrifugal forces.

Ideally, an icephobic surface should meet all the three criteria listed above, however, this type of surface has not been developed yet.^[^ ^43^ ^]^ Wettability plays a key role because it determines the ability of the surface to delay ice nucleation.^[^ ^75^ ^]^ It is important to note that while wettability can delay ice formation, this is a kinetic effect, meaning that ice will eventually form on the surface regardless. For this reason, wettability cannot be the sole parameter in designing an effective icephobic surface, as ice adhesion must also be considered.

The following paragraphs describe the state‐of‐the‐art of the most common strategies adopted in fabricating coatings for passive ice protection.^[^ ^9^, ^48^, ^86^ ^]^ All described coatings are categorized based on their anti‐icing working principles and their chemical nature into five main sections: coatings based on delaying ice nucleation and reducing ice adhesion through hydrophobicity (4.1); coatings that reduce ice adhesion through slippery effects (4.2); surfaces that reduce ice adhesion owing to the mismatch between the elastic modulus of ice and the surface material (4.3); coatings embedding phase change materials (PCMs) (4.4); and coatings based on the depression of the freezing point (4.5).

Finally, all the different strategies are summarized in two main tables: **Table** **1** collects the state‐of‐the‐art coatings based on the delaying of ice nucleation and the reduction of ice adhesion, while **Table** **2** reports the coatings based on the depression of the freezing point and the reduction of ice adhesion. A comparison of the main advantages and disadvantages of the proposed coatings is summarized in **Table** **3**.

**Table 1:** State‐of‐the‐art coatings based on the (super)hydrophobicity and the reduction of ice adhesion.

Typology	Coating	Substrate	CA [°]	CAH [°]/SA [°]	Ice adhesion [kPa]^a)^	Icing/de‐icing cycles	Refs.
Fluoro‐polymers	PTFE	Anodized aluminum	130	N.A.	30	N.A.	[87]
	PVDF	Reinforced glass fiber	156	2	N.A.	N.A.	[88]
	PVDF	Aluminum	168	2	10	N.A.	[89]
	PTFE	Aluminum, copper	162.8	3	N.A.	N.A.	[90]
	Fluorinated ethylene propylene	N.A.	110	20	N.A.	N.A.	[91]
	Teflon	Aluminum	165	3	N.A.	N.A.	[92]
	Fluoropolymers NPs, epoxy resin	Aluminum alloy	160	2	N.A.	N.A.	[93]
Poly‐siloxane elastomers	PDMS	N.A.	105	N.A.	22	N.A.	[94]
	Silica nanoparticles, polyacrylate binder, silicone resin	Aluminum	150	4	N.A.	N.A.	[95]
	Vinyl pendant polydimethylsiloxanes 4‐vinylphenylboronic acid, 20‐azobis(2‐methylpropionitrile), benzoil peroxide, 2,2,3,3,4,4,5,5,6,6,7,7‐dodecafluoroheptyl acrylate, silica, epoxy resin	Epoxide resin	150	5	53	N.A.	[96]
	1,1,3,3‐tetramethyldisiloxane	Glass	N.A.	N.A.	84.8	N.A.	[97]
	Methylhydrosiloxane, dimethylsiloxane copolymer, divinyl‐terminated poly‐siloxane	Aluminum	N.A.	N.A.	27.8	N.A.	[98]
	PDMS, 2‐ethylhexyl acrylate (EHA), 3,3,4,4,5,5,6,6,7,7,8,8,9,9,10,10,10‐ heptadecafluorodecyl methacrylate (TEMAc‐8) and 4‐vinyl pyridine (Vp)	Epoxy resin	113	N.A.	6	30	[99]
Metal surfaces	Perfluoropolyether	Laser patterned titanium	163	8	N.A.	16	[100]
	FAS‐17	Acid etched aluminum	158	4	20 N	N.A.	[101]
	Stearic acid	Oxygen peroxide etched aluminum	150	6	N.A.	N.A.	[102]
	FAS‐17	Acid, oxygen peroxide etched 1045 steel	155	N.A.	N.A.	N.A.	[103]
	Mecasurf	Laser patterned Aluminum AA204	166	10	N.A.	N.A.	[104]
	Mecasurf	Laser Titanium Ti64	169	7	N.A.	16	[104]
	Hexadecyltrimethoxy silane	CuCl_2_ etched Aluminum 1060	164	<10**°**	N.A.	N.A.	[105]
	FAS	Anodized copper	162	4	N.A.	50	[106]
	FAS	Basic etched Al 6061 alloy	150	<10**°**	33	N.A.	[107]
Organo‐gel	PMDS, Krytox	Glass	N.A.	N.A.	5.2	N.A.	[108]
	PDMS, silicon oil	Aluminum	N.A.	N.A.	6.5	30	[109]
	PDMS, silicon oil		N.A.	<5	5	12	[110]
SLIPS	Fluorinated silica, perfluoropolyether (Krytox)	Laser patterned titanium	131	2	17.95	10	[111]
	Fluorinated silica nanotube, perfluoropolyether (Krytox)	Glass	114	<10	17	24	[112]
	Porous polypirrole, tridecafluoro‐1,1,2,2‐ tetrahydrooctyl)trichlorosilane, perfluoropolyether (Krytox)	Aluminum	117	2	15.6	N.A.	[113]
	PTFE membrane, Krytox 103		110	7	35	N.A.	[114, 115]
	PDMS, silicon oil (100 cp)	N.A.	97	2	15	24	[116]
	Epoxy resin E‐51, silica microsphere, APTES, FAS, Krytox CBF‐100A	Aluminum	110	<10	10	20	[117]
	Carbonyl Iron NPs, PDMS, silicon oil (100 cp)	Aluminum	102	6	16	60	[118]
	Hydrophobic iron oxide NPs, cocoa oil	Hydrophobized anodized aluminum	135	<10	7.3	15	[119]
	Epoxy resin, Hydrophobic SiO_2_ NPs, Camelia oil	Aluminum	100	N.A.	<10	10	[120]
	Alumina, FAS, Krytox 105	Aluminum	119	9	N.A.	10	[121]
Sol‐gel derived surfaces	MTES 1H, 1H, 2H, 2H‐perfluorodecyltriethoxysilane Silica Glymo	Glass	170	<10	75	N.A.	[68]
	Epoxy resin, hydrophobic SiO_2_ NPs	Aluminum	113	N.A.	5	20	[122]
	Fluorinated silica and silica NPs	Glass	160	8	N.A.	10	[123]
	TEOS, Glymo, silica NPs, 1H, 1H, 2H, 2Hper fluorooctyltriethoxysilane	Glass	150	5	100	N.A.	[124]
	Titanate nanotubes, TEOS, Glymo, PFOTES	Glass	107	20	60	N.A.	[125]
	Zinc Oxide, dodecanthiol	304 stainless steels	150	5	N.A.	N.A.	[126]
	Silica NPs. PTFE	Soda lime glass	140	N.A.	N.A.	N.A.	[127]
	Alumina, Lauric acid	Aluminum	147.7	N.A.	0.8 N	N.A.	[128]
	ZnO, Silica NPs, Methyltrimethoxy silane	Q235 steel	150	3	N.A.	10	[129]
MOF	APTES UiO‐66 Silicon oil (500 CSt)	Glass	150	4	35	16	[130]
	ZIF‐8, perfluorooctyl silane	Copper	172	5	N.A.	30	[131]
	ZIF‐67 (Co), silicon oil	Copper (functionalized with dodecanthiol)	101	5	7.8 N	8	[132]
	UiO‐66 ‐NH_2_ + silicon oil	Polypropylene fabrics	102	N.A.	20	10	[133]
	Dopamine, UiO‐66‐COOH, 1 H,1 H,2 H,2 H‐Perfluorodecyltrichlorosilane	PU sponge	161	N.A.	N.A.	30	[134]
	Cu‐CAT‐1 + PDMS	Cu‐foil					[135]
	UiO‐66, Glymo, poly(methylhydrosiloxane MW = 7000)	γ irradiated PP	132	N.A.	9.4	30	[136]
	MOF‐MNS, 1H,1H,2H,2HPerfluorodecyltrimethoxysilane (PFTS)	Micronanostructured copper foil	150	N.A.	N.A.	20	[137]
	Epoxy resin, ZIF‐7@ZnG NPs, 1H,1H,2H,2H‐perfluorodecyltriethoxysilane (PFDS)	Q235 Carbon steel, glass	164	4	37	N.A.	[138]
LLS	chlorine‐terminated siloxane	Silicon, glass, and metals	106	1	26	10	[139]
	SYLGARD 184 Silicone Elastomer (PDMS)	PET covered with a layer of organic‐inorganic hybrid coating	105	30	99	N.A.	[140]
	Hydroxypropyl polydimethylsiloxane covalently attached to sol‐gel silica layer	Glass	106	N.A.	150	50	[141]

^a)^ In some case, ice adhesion is express in Newton, instead of kPa. When the value is expressed as a force, the unit of measure is reported in the table. In the other case, the unit of measure is kPa.

**Table 2:** State‐of‐the‐art coatings based on the depression of the freezing point and the reduction of ice adhesion.

Typology	Coating	Substrate	Freezing temperature	Ice adhesion [kPa]	Refs.
Hydrogel	Polyacrylic acid, propylene‐glycol, water	Stainless steel	N.A.	10	[27]
	PVA, ethylene glycol, water, borax	Copper	N.A.	<2	[142]
Electrolyte hydrogel	PVA, water solution of Fe_2_(SO_4_)_3_	Copper	−20		[143]
	Short‐chain chitosan, sodium citrate		−25		[144]
	Cotton cellulose, water solution CaCl_2_, ZnCl_2_		−70		[145, 146]
	PVA, NaCl water solution (20 w%)	Glass		≈0	[147]
Poly‐electrolyte	Polystyrene sulfonate polyelectrolyte	Textured Steel	−19.5		[148]
	Poly(3‐sulfopropyl methacrylate)	Gold	−21		[149]
	[(Methacryloyloxy)ethyl]trimethylammonium chloride	Glaas, ceramics, metals	−20		[150]
	Poly(sodium 4‐styrenesulfonate)/poly (allylamine hydrochloride), PSS/poly (diallyldimethylammonium chloride)	N.A.	−18		[151]
	[2‐(Methacryloyloxy)ethyl]‐ trimethylammonium chloride], [poly(3‐sulfopropyl methacrylate), poly(sodium methacrylate)]	Glass	N.A.		[152]
	Poly(vinylsulfonate, sodium) Poly(diallyldimethylammonium chloride)	Silicon wafer	N.A.		[153]

**Table 3:** Overview of pro and cons of all the mentioned approaches in paragraph 4.

Coating type	Ice protection mechanism	Pros	Cons
Fluorinated polymers^[^ ^87^, ^88^, ^89^, ^90^ ^]^	Low ice adhesion, hydrophobicity	Weak electrostatic interaction between ice and the surface. C‐F bonding increases the hydrophobicity of the surface.	Harmful to human health. Industries are banning and avoiding the usage of fluorinated compounds. High cost of the polymer.
Poly‐siloxane‐based viscoelastic coatings^[^ ^94^, ^154^, ^155^ ^]^	Low ice adhesion, hydrophobicity	Good adhesion vs. glass and silica substrates. High contact angle (CA) and sliding effect due to its viscoelasticity.	Weak mechanical durability Bad adhesion properties vs non‐glass/silica substrates
Metal surfaces^[^ ^100^, ^102^, ^104^ ^]^	Hydrophobicity (lotus leaf structure), low ice adhesion	Cost‐effective (chemical etching), superhydrophobic surface (SHS).	Expensive (laser texturing) The lack of mechanical durability. Anti‐icing properties when the pillar‐to‐pillar distance is smaller than a stable ice nucleus.
SLIPS^[^ ^156^, ^157^, ^158^ ^]^	Low ice adhesion, hydrophobicity	Low tilt angles and low hysteresis CAs. Defect‐free surface. Self‐repairing lubricant layer.	Lubricant leaching. Expensive Weak mechanical durability
Gels^[^ ^108^, ^159^ ^]^	Freezing point depression, low ice adhesion strength	Cost‐effective, nontoxic lubricant.	Limited applications scenario. Leaching of the liquid phase, Durability. The drastic change of properties below the melting point.
Polyelectrolyte brushes^[^ ^149^ ^]^	Depressing the freezing point, low ice adhesion	Avoiding the use of organic lubricants.	Expensive.
Sol‐Gel derived surfaces^[^ ^63^, ^68^, ^160^ ^]^	Depending on the synthetic approach	Cost‐effective synthesis. Tunable structures and introduction of ad‐hoc functional groups. High quality of the film. Possibilities of tuning morphology and roughness. Facile upscaling Hybrid coatings.	Weak to hydrolysis. The design of a superhydrophobic rough surface does not guarantee a Cassie–Baxter wettability state. High use of solvents.
MOF^[^ ^135^, ^161^ ^]^	Depend on the synthetic approach	Tunable properties. According to the synthesis condition, it is possible the design of hydrophobic MOF or SLIPS.	Few research papers on the topic. Mechanical properties have not been studied yet. Adhesion on a substrate. Lubricant diffusion into MOF channels has not been studied yet.
LLS^[^ ^139^, ^140^, ^141^, ^162^, ^163^, ^164^, ^165^ ^]^	Low ice adhesion	Low hysteresis CAs.	Few research papers on the topic. Adhesion on rough surfaces.

### 4.1 Icephobic Surfaces Based on Delaying Ice Nucleation and Reducing Ice Adhesion Through (Super)hydrophobicity

Delaying ice nucleation is associated with the energy barrier during heterogeneous ice nucleation. This barrier tends to increase when the hydrophobicity increases and the average roughness is smaller than the dimensions of the stable ice nuclei.^[^ ^47^ ^]^ It is important to note that the combined effects of surface roughness and wettability in achieving superhydrophobicity have been the main focus in the literature, while the individual contributions of wettability and roughness are less frequently addressed.^[^ ^166^ ^]^

The CA can be tuned by the introduction of functional groups that possess a low surface energy, such as fluoro‐containing groups or alkyl chains (**Table** **4**).

**Table 4:** Functional groups and their surface energy.^[^ ^161^ ^]^

Surface composition/surface	Surface energy [mNm^−1^]
─CF_3_	6–7
─CF_2_H	15
─CF_2_─CF_2_─	18
─CF_2_─CH_2_─	25
─CH_3_	20–24
─CH_2_─CH_2_─	31
─CH─(phenyl ring edge)	35
─CCl_2_─CH_2_─	40
MgO (100)	1200
Silicon (111)	1240

Inspired by the lotus leaves, SHSs have been designed by creating micronanostructures patterns on the surfaces and modifying the surface energy with low surface energy groups, which can enhance the anti‐icing properties of a surface. One of the main advantages of the lotus leaf structure is the nucleation in a convex site. Because of the reduced contact area, nucleation has a higher activation energy barrier. In addition, air trapped within the surface's roughness and the forming ice layer can cause stress due to the presence of an air thermal barrier, as air is a poor heat conductor (typical Cassie–Baxter mode).^[^ ^167^ ^]^ At low temperatures or when condensation occurs, water can infiltrate the roughness. A change from the Cassie–Baxter wetting state to a Wenzel one (“Cassie–Wenzel transition”)^[^ ^168^ ^]^ may happen with a drastic reduction of the anti‐icing properties. In the Cassie–Baxter state, air acts an insulator and crack initiator that is trapped between the surface roughness and water. In the Wenzel state, the water pressure is greater than air resistance, causing the surface roughness to be filled by water, which allows ice to nucleate and growth within the rough features.^[^ ^169^ ^]^ The “Cassie–Wenzel transition” does not occur when a droplet is gently poured onto the surface, but it is likely to happen when water impacts the surface or when a droplet is small and subject to vibrations. When the pressure at the water/air interface increases, the interface becomes curved. This pressure continues to rise until it reaches a limit value, which depends on the system. If exceeded, the trapped air layer collapses causing the transition from a Cassie–Baxter to a Wenzel wetting mode.^[^ ^168^ ^]^ This transition can be inhibited by tuning the microstructure of the surface. When the roughness of a textured surface is smaller than the radius of the smallest ice nuclei, water icing and the Cassie–Baxter wettability state can be maintained until its microstructure features are preserved.^[^ ^100^ ^]^

When the interaction between water and the surface is associated with a high energy, such as the case of a bouncing droplet over a surface, the idea that a hydrophobic surface can delay ice nucleation may not correspond to a real situation. On one hand, it is well‐known in the literature that the wettability properties of a surface can hinder the kinetics of ice formation. On the other hand, the effect of these properties on icing after a drop‐impact is less clear. Indeed, Kong and coworkers^[^ ^54^ ^]^ studied a set of aluminum surfaces with different wetting properties (hydrophilic aluminum, hydrophobic aluminum, and super hydrophobic aluminum). The findings revealed that at a temperature of −5 °C, water droplets freeze immediately upon impact on hydrophilic surfaces, freeze with a delay on hydrophobic surfaces and do not freeze on SHSs. However, when temperature is lowered to −15 °C, water freezes immediately without any rebound on all the surfaces tested.^[^ ^54^ ^]^ This suggests that, under conditions of impact icing, the wettability properties of the surface are not the primary factor in preventing ice formation.^[^ ^54^ ^]^

In addition to the inhibition of the nucleation, the surface can also promote a reduction of ice adhesion strength. Electrostatic forces, hydrogen bonding, Van der Waals (VdW) forces and mechanical adhesion are responsible of the ice adhesion strength.^[^ ^48^ ^]^ Electrostatic interactions can be evaluated using the dielectric constant of the material. For example, removing ice from a coating made of an oxide, such as, TiO_2_ (dielectric constant *ɛ* = 80), needs a large value of shear force (≈420 kPa) than the ice removal from a Teflon surface (ɛ = 2, ≈188 kPa).^[^ ^170^ ^]^ VdW interactions can be reduced by lowering the contact area between water and the surface, or by exploiting coatings with a less dense structure. Strategies such as lowering the amount of cross‐linker or adjusting the steric hindrance of the coating can effectively reduce the VdW forces. The introduction of low‐energy surface groups in the coating, such as hydrocarbon or fluoro‐compounds, can prevent the formation of hydrogen bonds with water due to the low polarizability of the C‐H and C‐F bond.^[^ ^171^ ^]^ Mechanical interlocking adhesion derives from the ice blockage by the geometry of the surface which decreases the efficiency of ice removal. Applying mechanical stresses can lead to the cracking of the ice structure.^[^ ^167^ ^]^

#### 4.1.1 Fluoropolymers

Fluoropolymer coatings are a feasible solution for effective ice protection. Fluoropolymers can be applied in various forms, such as ink or aqueous dispersion on mirror‐polished metals such as aluminum.^[^ ^172^ ^]^ Anodized aluminum substrates have been coated through sputtering of polymer‐silica composite materials^[^ ^173^ ^]^ and the fabrication of pillared fluorinated poly‐propylene sheet has been reported in literature.^[^ ^91^ ^]^ The anti‐icing properties can be attributed to the presence of the C‐F functional groups within the polymeric backbone and their low dielectric constant.^[^ ^173^ ^]^ The C‐F bonds contribute to an increased water CA and water repellency,^[^ ^159^ ^]^ enabling the final coating to reach hydrophobic properties.^[^ ^87^ ^]^ Furthermore, the low dielectric constant leads to a weak electrostatic interaction between polymers, such as poly(tetrafluoroethylene) (PTFE), and ice.^[^ ^87^ ^]^ Menini and coworkers functionalized an anodized aluminum surface with PTFE, resulting in a hydrophobic surface with a CA of 130°. This coating allowed for the removal of the ice deposit with a shear stress of 30 kPa. However, the coating material had low mechanical durability.^[^ ^87^ ^]^ Peng and coauthors developed a method to protect a reinforced glass fiber wind blade from ice formation by applying a super‐hydrophobic polyvinyldienefluoride (PVDF) coating. They sprayed the polymer with ammonium bicarbonate, which created a porous polymeric structure as ammonia was released and volatilized. This coating displayed a water CA of 156° and a sliding angle similar to that of the lotus leaf (≈2°). The super‐hydrophobicity of the surface was enhanced by both the effect of the ─CF_2_ functional group and the porous structure. As a result, the as‐prepared surface was protected from ice formation for 60 min at a fixed temperature of −10 °C. This success was attributed to the surface's ability to effectively remove water due to its sliding angle and CA.^[^ ^88^ ^]^

#### 4.1.2 Texturing of Metal Surfaces

Metals are typically characterized by hydrophilic surfaces. For examples, aluminum alloys show water CAs ranging from 60° to 80°. It is possible to modify these surfaces to transform them from hydrophilic to hydrophobic. One common approach involves texturing the metal surface, followed by functionalization to create a lotus‐like morphology.^[^ ^101^ ^]^ Several etchings have been tested for this purpose: oxygen peroxide, acids, and iron trichloride.^[^ ^34^ ^]^ However, a drawback of this strategy is the lack of precise control of the surface morphology. In contrast, direct laser texturing or lithography can achieve finer control, allowing the creation of pillared structures with specific pillar dimensions, pillar‐to‐pillar distance and depth.^[^ ^100^ ^]^ Common metals that can be textured include aluminum,^[^ ^28^ ^]^ titanium,^[^ ^100^ ^]^ copper,^[^ ^174^ ^]^ steel,^[^ ^148^ ^]^ and their respective alloys. Low surface energy groups are usually introduced on the surface using alkylsilanes or perfluoroalkysilanes. Recently, alternative methods such as siloxane functionalization^[^ ^101^ ^]^ or the greener modification by fatty acids have been explored due to the promising anti‐icing properties.^[^ ^102^ ^]^ The main limitation of nanostructured metal surfaces is their susceptibility to wear over time from abrasion, dust, and ice shards. The effectiveness of the surface is highly dependent on surface parameters, including surface roughness and pillar‐to‐pillar distance (for surfaces fabricated by laser texturing or lithography). The roughness needs to be optimized to guarantee a “Cassie–Baxter” state for incoming water and promote the detachment of ice through air‐induce crack propagation.^[^ ^100^ ^]^

Liu and coworkers^[^ ^101^ ^]^ investigated the icephobicity of several aluminum alloys that were chemical etched under acid conditions and functionalized with perfluoroalkylsilanes FAS 17. All the so‐prepared alloys were superhydrophobic, showing a lotus leaf like micronanostructure with a hierarchical roughness. They found that ice nucleation was inhibited when supercooled water approached a surface with minimal roughness, as this configuration minimized the water/surface contact area and reduced the heat transfer. Conversely, on rougher surfaces, water was trapped in the nanostructure, leading to rapid ice nucleation. Indeed, the most effective surface for reducing ice adhesion featured a dual size microstructure: a rectangular‐like microstructure of 0.5–10 µm and a large one ranging from 10 to 40 µm. The force required to remove ice from this surface was below 20 N. Larger spatial parameters, such as a hilly‐like micro morphology with a space ranging from 50 to 150 µm, required higher forces to remove ice (≈35 N). Small rough systems showed lower mechanical durability, which represents a strong limitation for their practical application.^[^ ^101^ ^]^ A similar trend was noticed by Soltis and coworkers, who coated a titanium surface with a titanium aluminum nitride coating. They found that the surface performed best with a roughness below 0.6 µm.^[^ ^175^ ^]^ Tong et al. prepared an aluminum alloy surface by chemical etching it with oxygen peroxide to create texture, followed by functionalization with stearic acid. The fabricated surface had a roughness of 4 µm, a CA of 150°, and a sliding angle of 6°, demonstrating the capability to delay the onset of nucleation by 34 min.^[^ ^102^ ^]^

#### 4.1.3 Superhydrophobic Surfaces Fabricated by Sol‐Gel

Sol‐gel synthesis is a versatile route to synthesize oxides and hybrid materials with specific and customized properties. This method is based on the hydrolysis and condensation reaction of alkoxide. When precursors present a non‐hydrolysable bond, it becomes possible to introduce functionalities within nanoparticles or thin film through co‐condensation and formation of a hybrid organic–inorganic network. Coatings formulated with tetraethyl orthosilicate (TEOS) and methyltriethoxysilane (MTES) can be engineered to achieve a flat hydrophobic surface with minimal CAH.^[^ ^33^ ^]^ This versatile method enables the incorporation of customized functionalities through co‐condensation, which may include alkyl chains,^[^ ^36^ ^]^ perfluoroalkyl chains,^[^ ^176^ ^]^ phenyl rings^[^ ^37^ ^]^ or amino,^[^ ^177^ ^]^ vinyl,^[^ ^178^ ^]^ and glycidyl groups.^[^ ^32^ ^]^ Given the inherent versatility, cost effectiveness, and ease of scalability for larger applications, this synthesis is attractive for industrial‐scale application. Sol‐gel coatings have been prepared by dip‐coating,^[^ ^36^ ^]^ spin coating, spray coating,^[^ ^63^ ^]^ on a wide range of substrates, including silicon, glass,^[^ ^63^ ^]^ metals such as aluminum^[^ ^36^ ^]^ and steel,^[^ ^129^ ^]^ and composite materials as carbon fiber reinforced epoxy resins.^[^ ^179^ ^]^

Despite the wide range of possibilities offered by the sol‐gel process, icephobic coatings have not been extensively studied, with only a few works available in literature. The modified functionalized silica prepared by Fu can be considered as the reference material for the icephobic properties of sol‐gel based coatings.^[^ ^63^, ^68^ ^]^ This coating consists of MTES and 1H, 1H, 2H, 2H‐perfluorodecyltriethoxysilane as a low surface energy component, nanosilica as filler to enhance surface roughness, and 3‐glycidyloxypropyl trimethoxysilane (GLYMO) to improve the adhesion to the substrate.^[^ ^63^ ^]^ The prepared coating was sprayed onto a glass surface, having a roughness of 1.8 µm and raising the CA to 163° with a Cassie–Baxter wetting mode at −10 °C.^[^ ^63^ ^]^ The amount of stress required for ice removal is influenced by the filler loading in the system. A minimum stress of 0.44 MPa was observed to remove ice from a system with a 5% filler content.^[^ ^68^ ^]^ Similarly, Wu et al. prepared a sol‐gel coating by spraying a solution of TEOS, GLYMO, 1H, 1H, 2H, 2H–perfluorooctyltriethoxysilane (PFOTES), and silica nanoparticles on glass slides and glass fiber reinforce epoxides. The resulting coating has a CA of 105°, sliding angle < 10° and roughness of 300 nm. Ice adhesion on the as‐prepared surfaces is reduced at 58 kPa at −15 °C.^[^ ^180^ ^]^

Carreno et al. sprayed a solution of TEOS‐GLYMO and commercial polyurethane paints over carbon fiber or glass fiber reinforced epoxides composite. The final coating had a water CA of 83° and a roughness of 5 µm and an ice adhesion value of 90 kPa at −15 °C.^[^ ^179^ ^]^

The properties of modified titania coatings were exploited to be applied in icephobic coatings. Unmodified titania is typically hydrophilic, due to the presence of −OH bonds on the surface when exposed to a water‐rich environment. Through an accurate surface functionalization, the hydroxyl groups can be substituted with more hydrophobic functionalities. Ayres et al. prepared titania particles from titanium isopropoxide and tripopylen glycol. The resulting coating displayed a glycerol ligand between two metal centers.^[^ ^181^ ^]^ Qi et al. introduced a methacrylic group in a titania particle by a functionalization with methacrylopropiltrimetoxysilane.^[^ ^182^ ^]^ The polymerization of a perfluorate methacrylate polymer and the as‐prepared particles conferred hydrophobic and icephobic properties to the composite material. The resulting material shows an icing delay of 25 min compared to the untreated titania.^[^ ^183^ ^]^

A limit of the applicability of sol‐gel based coatings in icephobic systems is the high rate of hydrolysis of silica in hybrid silica coating.^[^ ^160^ ^]^ A wide range of precursors was co‐deposited with TEOS, including MTES,^[^ ^36^ ^]^ phenyl‐triethoxysilane (PhTES),^[^ ^37^ ^]^ and *n*‐aminopropyl‐triethoxysilane.^[^ ^177^ ^]^ The extent of hydrolysis was found to be larger and faster in coatings with a higher ratio of TEOS. When the percentage of the precursor with hydrophobic functional groups increased, hydrolysis was hindered.^[^ ^160^ ^]^ Additionally, intramolecular reactions, such as acid‐base interactions between adjacent amino groups of the precursor and hydroxyl groups of silica, can facilitate hydrolysis. A faster degradation rate was observed when the coating was exposed to harsh conditions, such as elevated temperature in a water‐rich environment.^[^ ^37^ ^]^ To mitigate this detrimental process, a potential strategy is to cover the remained silanol groups with titania or zirconia. These two oxides are less soluble than silica in aqueous media and prevent the attack of water, thereby hindering hydrolysis.^[^ ^177^ ^]^

#### 4.1.4 Metal‐Organic Frameworks

Metal‐organic frameworks (MOF) are a class of ordered porous materials that are formed through self‐assembly, consisting of an inorganic center and an organic ligand. Although their properties depend on the nature of the ligand and the selected synthesis parameter, MOFs are generally crystalline porous materials with large surface area and well‐defined topology, good thermal and chemical stability. MOF thin films can be prepared by spraying^[^ ^184^ ^]^ or dip‐coating^[^ ^185^ ^]^ a MOF solution on the surface on a wide range of substrates such as glass, and metals.^[^ ^138^ ^]^ Alternatively, MOF can growth directly on the metallic substrate after surface oxidation of the bare substrate.^[^ ^186^ ^]^

Recently, two main synthetic approaches have been reported for the synthesis of hydrophobic MOF: the introduction of hydrophobic groups branched to the ligand structure or a post‐synthesis functionalization with hydrophobic groups. They are both successful, but the presence of bulky groups might decrease the accessible surface area of the porous system.^[^ ^161^ ^]^ Canivet and coworkers reported the post‐synthesis functionalization of ZIF‐90, which displayed an aldehydic functionality that can react with a primary amine, such as dodecylamine, on the surface.^[^ ^187^ ^]^ Chen and coworkers developed a hydrophobic MOF through the self‐assembly of a fluorinate biphenyl ligand around a copper cation, achieving a CA of approximately 135°.^[^ ^188^ ^]^

Ice protection is a new application for this family of compounds, and currently, only a few studies address the topic. Zhu et al. sprayed ZIF‐7 nanoparticles on glass and carbon steel, which were subsequently functionalized with 1H,1H,2H,2H‐perfluorodecyltriethoxysilane. An epoxy primer layer was adopted to improve the adhesion of the particles. The resulting coating achieved a CA of 164° and a sliding angle of 4°. Thanks to the air trapped, water droplets have a delay of 150 s at −20 °C and ice adhesion was reduced to 37 kPa .^[^ ^138^ ^]^ Chen et al. spray coated a ZIF‐8 PFOTES composite on a Q235 steel coated with an epoxy resin as primer layer. The spray coating created a microscale roughness with pit protuberances larger than 10 µm on the smooth steel. The combination of roughness and hydrophobic groups of PFOTES conferred a water CA of 168° and a sliding angle of 2°. The coating prevented frost formation in a refrigerator at −20 °C for 2 h and could rebound a water droplet off from the surface before freezing.^[^ ^184^ ^]^

### 4.2 Reducing Ice Adhesion by Slippery Effects

An alternative approach to reducing ice adhesion and promoting ice detachment involves applying coating with slippery effects.^[^ ^189^ ^]^ The following section described the slippery liquid infused surfaces (SLIPS) and the liquid‐like surfaces (LLS),^[^ ^162^ ^]^ also known as slippery covalently attached liquid‐like surfaces (SCALS).^[^ ^163^ ^]^ In addition to the reduction of ice adhesion, these surfaces can delay ice nucleation because of their wettability.

#### 4.2.1 Slippery Liquid Infused Surfaces

Ice removal or ice delay properties of a SHS are less effective when the latter is exposed to a wet atmosphere. Trapped air cannot overcome water pressure and the liquid can diffuse throughout the surface roughness. A solution to bypass the limitation imposed by humidity was found in nature, studying the Nepenthes pitcher plants. Observing this plant, the idea of SLIPS aroused.^[^ ^114^ ^]^ These plants exploit the microstructure of the leaf to block an intermediary liquid that works as a repellent layer. Owing to the low CAH (<2.5°) and a smooth surface with a roughness around 1 nm, the leaf shows interesting hydrophobic and icephobic properties.^[^ ^114^ ^]^

A SLIPS consists in a porous host surface that traps a lubricant. To display anti‐icing properties, the system must satisfy three main criteria:^[^ ^114^ ^]^

1. The lubricant must wick inside the porosity, wet it, and stably adhere to the substrate.
2. The solid must be preferentially wetted by the lubricant and the repelled liquid should have no interaction with the host matrix.
3. The lubricant and the repelled liquid must be immiscible. The two liquids are considered miscible if the relative solubility is less than 500 parts per thousand by weight.^[^ ^156^ ^]^

Criteria 2 states that the surface energy of the lubricant/solid interface is lower than the surface energy of the immiscible liquid/solid interface. The freezing point of the lubricating liquid must be far below the freezing point of water, and the lubricant must be in the liquid state at the temperature the SLIPS system is supposed to work. The lubricant must not be a volatile compound and must have a low surface tension to win air resistance and wet all the matrix. When this condition is satisfied, the resulting surface is a flat defect‐free surface with low hysteresis CA and low sliding angle, providing extraordinary icephobicity to the material. SLIPS surfaces show promising self‐healing properties due to the spontaneous regeneration of the lubricant layer, which derives from the capillary forces moving the liquid from the inside space to the void outer voids.^[^ ^114^, ^156^ ^]^ However, the weak resistance to abrasion, shear forces, and the high cost constitute a limitation to an extensive application of this approach.

When the synthesis is concerned, a three‐step process is usually adopted: 1) fabrication of a rough surface in which the capillary force can retain the lubricant, 2) chemical functionalization of the surface to improve the affinity of surface and lubricant, 3) infusion of a lubricant layer.^[^ ^190^ ^]^

Chen et al. have proposed an approach for a rational design of SLIPS.^[^ ^156^ ^]^ In all the considered cases, the lubricant forms a stable layer over the surface when it is blocked by a chemical or physical barrier resulting in a discontinuous lubricant layer. A chemical barrier may be a π electrons wall,^[^ ^191^ ^]^ or a chemical bond between a functional group in the surface and the lubricant.^[^ ^136^ ^]^ A physical barrier can derive by the creation of channels, or porosity, connected (open cell) or isolated (close cell), where the lubricant is retained by capillary forces. A tridimensional porous pattern allows the storage of the lubricant in the channel system, and the self‐healing property of the surface is enhanced.^[^ ^156^ ^]^

**Figure 5:**
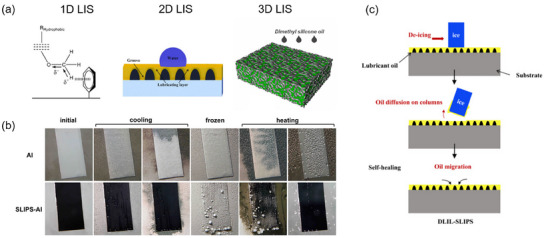
a) General classification of SLIPS:1D, lubricant retained by electrostatic interactions (Reprinted with permission.^[^ ^191^ ^]^ Copyright 2016, WILEY‐VCH Verlag GmbH & Co.); 2D, lubricant retained inside grooves of a textured surface by capillary forces (Adapted with permission^[^ ^111^ ^]^ Copyright 2023, Elsevier); 3D, lubricant retained by capillary forces inside the pore system of a porous solid. Adapted with permission.^[^ ^192^ ^]^ Copyright 2021, Elsevier. b) Icephobic behavior of a SLIPS on aluminum. Reprinted with permission.^[^ ^113^ ^]^ Copyright 2012, American Chemical Society. c) De‐icing mechanism of a LIS. Below an ice crystal, an oil layer causes a reduced adhesion and a roll‐off of the ice crystal. Reprinted with permission.^[^ ^111^ ^]^ Copyright 2023, Elsevier.

According to the microstructure, SLIPS can be classified as 1D, 2D, or 3D SLIPS (**Figure** **5**a).^[^ ^157^ ^]^ The first case consists in the retention of the lubricant by a chemical barrier due to the insertion of specific functional groups that can anchor the repellent liquid to the flat surface. The second consists in a patterned surface with grooves that can retain a lubricant by capillary forces. The third case is represented by porous materials that can trap the lubricant in their inner pores and cavity system.^[^ ^157^ ^]^

1D SLIPS can be prepared by the condensation of functionalized silane precursors by the sol‐gel approach. Tenjimbayashi and coworkers prepared a surface with enhanced capability of retaining the layer of lubricant leveraging the combination of TEOS and PhTES. A flat surface with a rich presence of an extended π electron density successfully retained a lubricant layer of triethoxydecylsilane. Due to the attractive effect of oxygen, the C‐H bond shows a positive partial charge that can interact with the π electron.^[^ ^191^ ^]^

Alternatively, exploiting lithography, 2D‐SLIPS can be designed by patterning the surface with microchannels where the lubricant is trapped by capillary forces. Zheng et al. patterned a titanium alloy coated with fluoro‐functionalized silica by a laser. The resulting surface consisted of a grooves array with a spatial period of 14 µm, infused by a perfluoropolyether.^[^ ^111^ ^]^ The structure of 3D‐SLIPS can be obtained by the layer‐by‐layer deposition of polymers, by the template synthesis of hollow nanoparticles, or by the ordered self‐assembly of porous materials. Considering a 3D porous material, most of the state‐of‐the art porous surfaces are open cells, an interconnected series of channels, such as mesoporous silica, filled by a lubricant. Recently, closed cell porous surfaces have been taken into consideration, due to improved mechanical performances.^[^ ^112^, ^156^, ^193^ ^]^

One of the first attempts was the synthesis of a porous polymeric matrix infused with a fluorinated liquid.^[^ ^114^ ^]^ This surface reduces the adhesion strength of ice below 16 kPa. A similar result was achieved by Pokroy and coworker. They prepared an ordered PMDS nanospots functionalized with a low surface energy perfluoroalkyl silane and a low surface tension perfluorinated liquid as lubricant was adopted to the improved chemical affinity of fluorinated functional groups of the substrate and the lubricant.^[^ ^194^ ^]^

The choice of the lubricant is a crucial parameter. It must not only respect the criteria above‐mentioned, but it must also consider the effect of lubricant leaching on human health and the environment. Perfluoropolyether and silicon oil are water repellent liquids characterized by a unique inertness. At room or environmental temperature, they are not degradable by any natural pathway and they can accumulate in living creatures and interact with biological systems.^[^ ^157^ ^]^ Nowadays, fatty acids and natural oil, such as coconut or olive oil, are emerging materials for the substitution of environment unfriendly liquids.^[^ ^157^ ^]^ However, the performance of natural oils has not been deeply studied yet.

Because of the reduced ice adhesion on the surface, SLIPS have been studied as anti‐icing materials. Kim et al. electroplated polypyrrole on aluminum, following fluorination by tridecafluoro‐1,1,2,2‐tetrahydrooctyl)trichlorosilane. The as‐prepared surface displayed a 2 µm roughness with globular‐shaped polypyrole particles. The capillary forces of the surface retained a lubricant layer of 8–10 µm composed of the Krytox 100 perfluoropolyether, which led to an ACA of 117° and a CA hysteresis of 2°. Ice cylinders were removed by a shear strength of 15.6 kPa at −10 °C.^[^ ^113^ ^]^ Zhang et al. prepared a SLIPS surface by spraying hollow tube silica on an etched glass substrate. To facilitate the impregnation of the Krytox, lubricant into the tubes, the silica structures was functionalized with heptadecafluoro‐1,1,2,2‐tetradecyltrimethoxysilane. The as‐prepared porous surface consisted of pore walls of 30 nm and hollow diameters of 1 µm. The freezing of a water droplet was observed after 2352 s, instead of the 84 s of the bare glass. Ice adhesion was 17 kPa and remained a constant value after 24 icing/de‐icing cycles.^[^ ^112^ ^]^

Zhu et al. prepared a rough surface by spraying silica nanoparticles on an epoxy coated aluminum substrate, infiltrated with silicon oil. To increase the affinity with the silicon oil lubricant, alkylic groups were introduced on the surface by the functionalization with hexadecyltrimethoxysilane. The resulting coating showed a roughness of 4.45 µm, a CA of 110° and an ice adhesion force of 6 kPa.^[^ ^120^ ^]^ Alternatively, camelia oil can be adopted as the lubricant layer. In this case, stearic acid increased the affinity between the nanoparticles and the lubricant layer. With a surface roughness of 3.14 µm and CA of 100°, the coating performance toward ice adhesion was 14 kPa.^[^ ^120^ ^]^ In addition to rough coatings, porous materials can also retain lubricants owing to the capillary forces. The large extension of channels, combined with their pore opening, makes MOF attractive for this purpose. In the MOF system, the pore opening and the functionalities of the organic linkers play a significative role in the retention of the lubricant. The pore opening should be larger than the lubricant diameter for a durable lubricant retention.^[^ ^195^ ^]^ Singh et al. coated a glass substrate with a SLIPS based on UiO‐66.^[^ ^130^ ^]^ UiO‐66 is a MOF with a Zr metal center and a functionalized 1,4‐benzene‐dicarboxylate ligand. According to Kandiah´s procedure,^[^ ^196^ ^]^ the resulting materials showed a crystalline mesoporous structure with a specific surface area of circa 1200 m^2^ g^−1^,^[^ ^196^ ^]^ good thermal resistance up to 300 °C, and good chemical resistance in acidic media.^[^ ^196^ ^]^ The UiO‐66 coating was formed through self‐assembly on a pre‐functionalized surface. A chemical bond between the amino‐functionalized glass and the acid functionality of the linker enhanced the chemical adhesion of the MOF to the substrate. The as‐prepared coating showed a pore opening of 6 Å, not sufficient to let the lubricant flow inside the inner porosity of the MOF.^[^ ^195^ ^]^ The silicon oil (500 CSt,^[^ ^130^ ^]^ diameter of 5.9 Å^[^ ^195^ ^]^) was retained only by the roughness created by MOF clusters grown on the substrate. The coating was 165 nm thick with a root mean square roughness of 80 nm, CA > 150° and a hysteresis of 4°. Such value of CA was maintained after 50 icing/de‐icing cycles. The adhesion strength of ice on this surface was 35 kPa and the performance was constant for 11 icing/de‐icing cycles.^[^ ^130^ ^]^

A further step in the SLIP research area would aim to design MOF coatings with pore opening large enough to promote the penetration of the lubricant in the channels system.

#### 4.2.2 Liquid‐Like Surfaces

A different strategy to achieve nonsticky surfaces with anti‐icing performances is represented by the LLS.^[^ ^162^, ^163^ ^]^ These surfaces consist of a thin polymer layer (1–5 nm) characterized by a low CAH (<5°). The low CAH is ascribable to the mobility of the flexible tethered molecules, which is correlated to the length of the polymer chains and the grafting density.^[^ ^164^ ^]^ The slippery properties of these surfaces reduce the adhesion of different solids, including ice, similar to SLIPS. Generally, LLS are fabricated by grafting PDMS chains to a surface. Polyethylene glycole (PEG), short‐chain alkanes, and perfluoro chains LLS are also reported.^[^ ^165^ ^]^ The main limitations of LLS concern their durability and the achievement of liquid‐like properties on a real practical surface rather than silicon wafers. Khatir et al. propose a two‐step approach to fabricate PDMS liquid‐like molecules on rough substrates (including metals, papers, and ceramics). A preliminary silica layer was deposited on the substrate, making the surface silicon wafer‐like smooth, followed by the vapor grafting of chlorosiloxane.^[^ ^197^ ^]^

Currently, only a few works deal with the practical application of LLS as an anti‐icing or de‐icing surface. Zhang et al.^[^ ^139^ ^]^ fabricated PDMS quasi‐liquid surfaces on various substrates (silicon, glass, aluminum) by one‐step self‐catalyzed grafting. The measured thickness was 30 nm (thicker than the general range defined for LLSs), and the reported water CAH was 1° (on silicon). These surfaces display a substantial reduction of the adhesion force between ice and substrate (26.1 kPa compared to 275.8 kPa of bare glass) and a durability of up to 10 cycles of icing/de‐icing. Li et al.^[^ ^140^ ^]^ grafted PDMS brushes on an organic–inorganic hybrid photothermal coating made of polypyrrole nanoparticles embedded in a silica layer. The ice adhesion strength was measured to be 98.7 kPa, four times lower than the bare photothermal hybrid system (460.7 kPa). In addition, owing to the photothermal properties, the coating favors the melting of ice under sun irradiation. Loop‐like PDMS chains with liquid‐like properties were grafted on silica NPs layers by two‐step dipping.^[^ ^141^ ^]^ Due to the mobility of PDMS chains and the low surface tension, the coatings showed anti‐icing and ice‐delaying properties. The freezing time on the hybrid film was prolonged to 1207 s, which was seven times longer than that on the glass surface (170 s). Low ice adhesion strength was maintained during 50 icing/deicing cycles.

### 4.3 Reducing Ice Adhesion by the Mismatch Between the Elastic Moduli of Ice and the Coating

The last proposed mechanism for reducing ice adhesion is based on the mismatch between the elastic moduli of ice (Young modulus in the range of 0.3–3.6 GPa) and of a soft material (Young modulus < 10 MPa).^[^ ^198^ ^]^ A group of coatings that successfully develop the above‐mentioned working principle are PDMS coatings and its homologues. When a rigid mass such as ice impacts a material with a different elastic modulus, cavities are created along the low‐elastic modulus side of the interface. Around the cavity, localized stress generates cracks which propagate on the interface and detach the mass—or ice—from the coating material.^[^ ^199^ ^]^ This typology of ice removal mechanism is known as deformation incompatibility.^[^ ^198^ ^]^ The elastic modulus of the soft coating can be lowered by reducing the cross‐linking of the polymeric network. The un‐cross‐linked chains promote the mobility of the ice‐substrate interface promoting a fast propagation of the cracks. However, soft polymers show low mechanical durability. A large grade of cross‐linking or the blending with inorganic particles causes both: the enhancement of the mechanical properties and a reduction in the anti‐icing performance of the polymer.^[^ ^198^ ^]^

A further class of coatings based on this principle is represented by organo‐gels. These materials combine the deformation incompatibly typical of a soft material, and the slippery effects of liquid‐infused surfaces.^[^ ^159^ ^]^

#### 4.3.1 Poly‐Siloxane Based Viscoelastic Coatings

Poly‐siloxanes consist of the repetition of a siloxane base structure with suitable functional groups. Their icephobic properties derive from the high CA, due to their nonpolar functionalities bounded to the Si center, and from their viscoelastic properties. Ibáñez‐Ibáñez and coworkers^[^ ^94^ ^]^ studied the adhesion of ice over a PDMS base surface. They discovered an ice block can be detached by a shear force of 22 kPa because of the stress accumulation at the ice/PDMS interface. In this system, ice detachment from the elastic surface is caused by interfacial cavitation, which is responsible for ice bouncing off the surface without any slippage.^[^ ^94^ ^]^ This class of coating materials shows good adhesion to glass/silica‐based materials, but the adhesive properties to other substrates are weaker, as well as the mechanical durability.^[^ ^9^ ^]^ To increase the binding strength to the substrates, such as metals, a binder can be attached to the siloxane‐based polymer.^[^ ^96^ ^]^ Metal oxides such as TiO_2_ and ZnO allow the formation of a lotus leaf‐like structure, which improves the hydrophobicity of the polymeric matrix. However, the dispersion of metal oxide nanoparticles in a polymeric matrix might lead to an inhomogeneity property of the coating layer. Polysiloxane elastomers are usually spin‐coated,^[^ ^94^ ^]^ sprayed,^[^ ^200^ ^]^ or grafted to the substrate,^[^ ^99^ ^]^ followed by a UV curing to balance the mechanical and the anti‐icing properties of the final coating.^[^ ^94^ ^]^ The polymeric matrix may be degraded if the composite material is spray‐coated at high temperature or prepared in severe conditions, leading to drastic reductions in the anti‐icing properties.^[^ ^201^ ^]^

Liang et al. fabricated a silica/fluorine‐functionalized PDMS composite by curing an epoxy resin and spraying the silica/PDMS blend on a glass substrate. The as‐prepared coating showed a CA of 156° and a thickness of 135 µm. Particles of 40 nm created a surface roughness which promotes superhydrophobicity. The coating was able to delay ice nucleation up to 2500 s and to reduce ice adhesion to 73 kPa.^[^ ^96^ ^]^

PDMS was also adopted as an additive in sol‐gel synthesis.^[^ ^94^ ^]^ Sobhani and coworkers observed that fillers such as silica improved the mechanical durability of the coatings. They fabricated a silica/PDMS composite coating on aluminum by a film applicator, starting by prehydrolyzed TEOS and hydroxyl terminated PDMS. The as‐prepared film had a thickness of 500 µm and a CA of 117°. The silica nanoparticles on the surface had a size of 125–250 nm. They successfully managed to improve the mechanical durability by increasing the precipitation of silica, resulting in an ice adhesion of 33 kPa for a silica loading of 20%. However, ice adhesion increased after five icing/deicing cycles and with the silica nanoparticles loading.^[^ ^122^ ^]^

#### 4.3.2 Organo‐Gels

**Figure 6:**
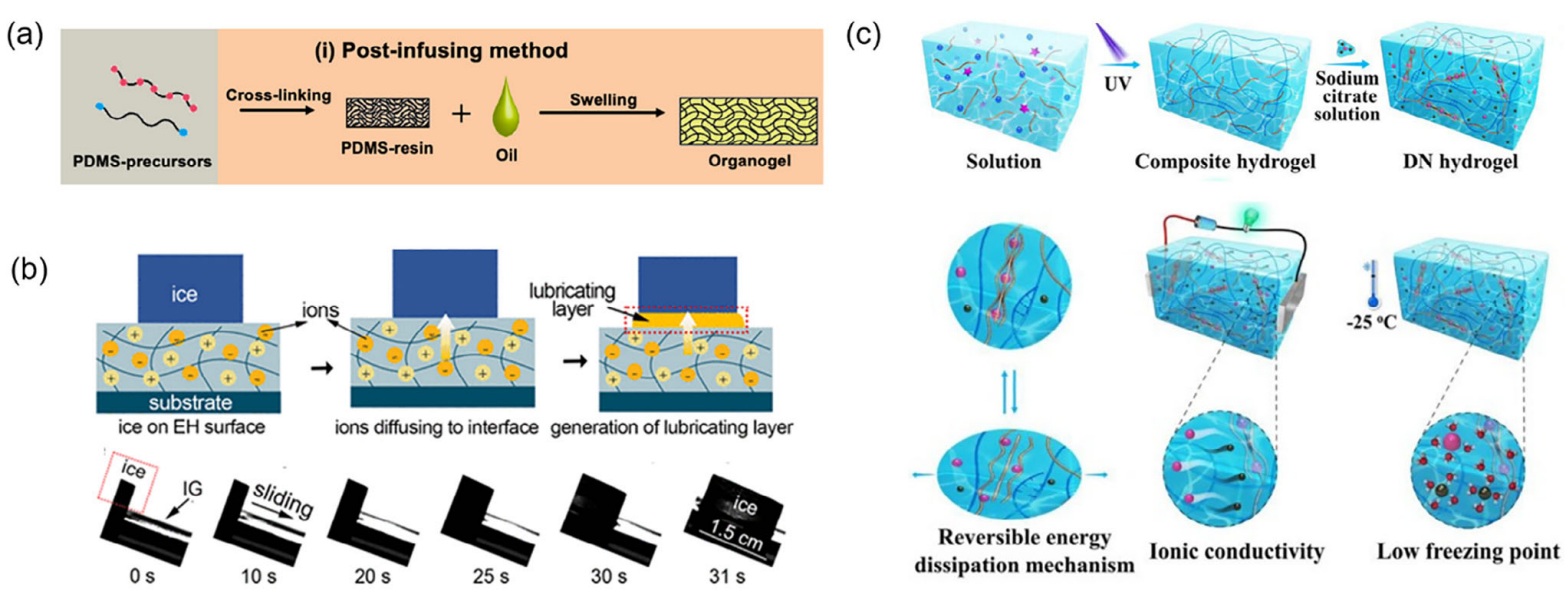
Gels preparation and their anti‐icing mechanism. a) Preparation of an organo‐gel. Oil is infused in a polymeric matrix. Adapted with permission.^[^ ^110^ ^]^ Copyright 2021, American Chemical Society. b) Mechanism of anti‐icing of an electrolyte‐hydrogel. Ice starts melting due to the freezing point depression of a salt solution. Melted ice forms a lubricant layer that helps the removal of the ice crystal, enhancing the sliding effect of the gel. Reproduced with permission.^[^ ^147^ ^]^ Copyright 2020, American Chemical Society. c) Preparation of an electrolyte hydrogel. A salt solution is trapped by a polymeric chain. Ions contributed to mechanical performances (through partial cross‐linking of polymeric network), ionic conductivity, and low‐temperature tolerance. Reprinted with permission.^[^ ^144^ ^]^ Copyright 2020, Elsevier.

Gels are a promising new class of coating materials with icephobic properties (**Figure** **6**a). Gels consist of a liquid phase dispersed in a solid cross‐linked one and show similarities to both SLIPS and elastomers, owing to the presence of a retained liquid layer and to the soft nature of the coating, respectively. According to the trapped liquid nature, gels can be classified as organo‐gels, hydrogel, and iono‐gels. Gels are usually prepared by casting a polymer precursors solution with the liquid phase on a substrate as glass,^[^ ^110^ ^]^ silicon, and metals^[^ ^202^ ^]^ as aluminum.^[^ ^109^ ^]^ PDMS,^[^ ^108^, ^110^, ^203^ ^]^ polyvinyl alcohol,^[^ ^142^, ^143^ ^]^ and methacrylate derivates^[^ ^202^ ^]^ are the most common polymers which retain the liquid phase. There are two main approaches^[^ ^202^ ^]^ to trap the liquid phase within the polymeric chains: the curing of the polymeric solution in the presence of a cross‐linker^[^ ^109^ ^]^ or the liquid infusion following the synthesis of the polymeric network. The former approach takes advantage of the one‐step formation of the gel by polymerizing and cross‐linking a precursor in the target liquid solution. Within this approach, the choice of the liquid phase is limited by the solubility in the final polymer. The latter takes advantage of a wide range infusion of organic liquids in the polymeric matrix and the limit is the two‐step synthesis of the final material.^[^ ^202^ ^]^ In this paragraph, only the anti‐icing performance of organo‐gels is reported, while hydrogel, polyelectrolyte hydrogel, and iono‐gel are described in detail in Section 4.5.1, to better consider the different mechanism of ice protection involved.

The liquid phase in organo‐gels is usually an organic solvent as alkanes and paraffines,^[^ ^110^ ^]^ but lubricants as silicon oils^[^ ^110^ ^]^ and perfluoroalkanes^[^ ^203^ ^]^ can be adopted too. The mismatch between the elastic moduli of the organic liquid and the ice crystal is responsible for ice detachment.^[^ ^144^ ^]^ While ice is growing, air is trapped between ice and the liquid layer. These air cavities diffuse over the surface and, if the latter is tilted, ice microcolumns slid off the surface.^[^ ^159^ ^]^ In addition, the organic liquid layer acts as the lubricant surface of SLIPS. The cross‐linking reaction increases the free energy of mixing, so liquid is spontaneously released to regenerate a flat liquid layer with self‐healing property. However, leaching of the organic solvent in the environment is a considerable limitation that was not yet overcame.^[^ ^159^ ^]^

Beemer and coworkers developed a PMDS based organo‐gel. The polymeric phase was prepared by the hydrosilylation reaction of the vinyl terminated PDMS and the hydride terminated PDMS in the trimethyl terminated PDMS as solvent, resulting in the retention of the solvent during the formation of the polymeric network. The coating, having a thickness of 1400 µm, and a roughness of 0.05 µm, showed low ice adhesion, allowing detachment of ice though the application of a shear stress of 5.2 kPa. The property was maintained up to 20 cycles.^[^ ^108^ ^]^ Alternatively, Sandhu et al. infused a wax and perfluoroalkanes into a cured PDMS matrix. The CA of the coating was 97°, the film thickness 200 µm, and the ice adhesion was 19.7 kPa.^[^ ^203^ ^]^

### 4.4 Improving Icephobic Properties by the Introduction of Phase Change Materials in the Coating System

PCMs are compounds which release latent heat as a response of their solidification. A good thermal diffusion of the released heat guarantees a fast warming of the coating and the maximization of PCMs effects in the anti‐icing field.^[^ ^204^, ^205^ ^]^ Between all the common typology of PCMs, such as hydrated salts or esters, fatty acids, and alcohols,^[^ ^205^, ^206^ ^]^ the most relevant PCMs for practical anti‐icing applications are n‐alkanes,^[^ ^207^, ^208^ ^]^ owing to their melting point close to the freezing temperature of the water and a latent heat in the range 146–271 kJ kg^−1^.^[^ ^209^ ^]^

A promising approach to incorporate PCMs into a coating is the encapsulation of the active chemicals (core) in a chemical inert shell. This approach has the following advantages: the capsules are simple to be handled and processed, they retain the active chemicals and enhance the stability of the system toward the surrounding environment by preventing any chemical interaction or the dissolution of the active phase into the coating.^[^ ^204^ ^]^ The encapsulating shell could be made of polymers,^[^ ^204^, ^210^ ^]^ as for example urea‐formaldehyde shells prepared by in situ polymerization in emulsion.^[^ ^208^ ^]^ The shape and the size of the capsules have a large impact on the PCMs heat exchange capability and stability.^[^ ^210^, ^211^ ^]^ The anti‐icing properties of the coatings are drastically reduced when the PCMs dissolve or react in the surrounding environment or leach out from the coating they are incorporated into. The mass loss of PCMs is the most relevant degradation mechanism of the as‐fabricated surfaces.

PCMs have been incorporated in siloxane coatings.^[^ ^207^, ^208^ ^]^ When the latent heat of PCMs is released, the coating temperature bounces up the freezing temperature of the water, delaying the reaching of the optimal temperature for water‐to‐ice transition, and as a consequence, avoiding the complete freezing of the droplet. The effectiveness of this approach is strongly related to the capability of heat conduction of the surface.^[^ ^204^ ^]^ The combination of the release of the latent heat with the nanoroughness due to PCMs incorporation,^[^ ^207^ ^]^ and the hydrophobicity of the coating matrix creates a Cassie–Baxter wetting mode, which facilitate the removal of water before freezing.^[^ ^204^ ^]^ On a PDMS surface filled with PCMs, the delay time of a water droplet increased from 181s (on the bare PDMS) to 1196 s.^[^ ^207^ ^]^

The reduction of ice adhesion can be achieved by fracture propagation induced by volume changes,^[^ ^207^ ^]^ or by the presence of a liquid‐like layer, which confers slippery effects to the surface.^[^ ^208^ ^]^ The first approach is based on the mismatch between the volume reduction in the cold region of the surface, and the volume expansion in the heated area caused by the release of the latent heat from PCMs. The mismatch generates local fractures that propagate when an external stress is applied on the surface.^[^ ^207^ ^]^ In the second approach, the latent heat released during the phase change is transferred from the capsules to the ice, resulting in a partial melting of the ice crystals formed on the surface. The partially molten ice created a quasi‐liquid layer at the coating/ice interface, which facilitates the slippage of the ice. Shamshiri et al. managed to stabilize the quasi‐liquid layer by blending the icephobic PEG‐PDMS copolymer with hydrophilic groups. The ice adhesion on the final blend was reduced from the 380 to 96 kPa.^[^ ^208^ ^]^ Additionally, the air, trapped within the nanoroughness created by the encapsulated PCMs, reduces the ice adhesion strength as the case of the SHSs.^[^ ^204^ ^]^

### 4.5 Icephobic Surfaces Based on the Reduction of the Freezing Point and Low Ice Adhesion

Another strategy for the creation of icephobic surfaces concerns the depression of the freezing point while simultaneously lowering the ice adhesion strength. According to the phase transition theory, solid water and liquid water are in equilibrium at defined conditions of temperature and pressure. The values of pressure and temperature that allow the co‐existence of water and ice define the “freezing point” of water.^[^ ^212^ ^]^ In analogy of what happens in a solution, in which the freezing point can be tuned by decreasing the vapor pressure of the pure solvent through the addition of a solute, a surface can also induce a variation of the water vapor pressure. Indeed, the freezing temperature is depressed when water is confined into small pores (the so‐called Gibbs–Thompson effect),^[^ ^213^ ^]^ or due to trapped molecules (such as ions) behaving as a solute in solution. The addition of 30 m/m% calcium chloride to a water solution depresses the freeing point down to −52 °C.^[^ ^145^ ^]^ In this scenario, hydrophilic surfaces such as electrolyte or polyelectrolyte hydrogel and polyelectrolyte‐decorated surfaces can also show icephobic properties. The freezing point can be depressed by controlling the water‐to‐ice transition state during solidification. To complete the phase change, water molecules form a hexagonal adduct similar to the ice structure by dipole–dipole interactions. Ionic groups can disrupt the transition state by electrostatic interaction between water dipoles and the charge of the ions.^[^ ^214^ ^]^ Water molecules are coordinated to the ionic group without assuming the spatial arrangement to change phase, resulting in the depression of the freezing point.^[^ ^149^ ^]^

#### 4.5.1 Electrolyte Hydrogels, Polyelectrolyte Gels, and Iono‐Gels

Hydrogels are gels with water as the liquid phase. As‐pointed out in paragraph 4.3.2, hydrogel can be prepared by one step polymerization of a water compatible polymer starting from an aqueous solution,^[^ ^145^ ^]^ or by a two‐step approach.^[^ ^143^ ^]^ Polyvinyl alcohol,^[^ ^143^ ^]^ polyacrylic acid,^[^ ^27^ ^]^ and polyacrylamide‐alginate^[^ ^145^ ^]^ are common polymer backbone, as their polar group can successfully retain water in the polymer chains. A pure hydrogel shows no icephobicity, but trapping a salt solution, instead of pure water, into a polyvinyl alcohol polymer improves the anti‐icing properties due to the depression of the freezing point (up to –20 °C) (Figure 6c).^[^ ^147^, ^159^, ^215^ ^]^ When ice grows on the electrolyte hydrogel coating, the diffusion of ions from the coating melts the ice at the coating/ice interface, creating a “non‐freezable” water layer which induces slippery effects and weakens ice adhesion to the surface (Figure 6b).^[^ ^159^ ^]^ Similarly to salt solutions, glycols can depress the freezing point of water. As example, Yao and coworkers coated a cotton fabric with a polyacrylic acid hydrogel. The fabrication of the hydrogel followed a two step‐approach, where first polyacrylic acid was polymerized in the presence of a cross‐linker inside a mold containing the textile. After that, propylene glycol was introduced by soaking the composite in a water‐propylene glycol solution 60 m/m% of propylene glycol. Ice was found on the surface of the hydrogel‐fabric composite after 1 h at −30 °C.^[^ ^27^ ^]^

Inspired by anti‐icing proteins,^[^ ^216^ ^]^ a polymeric matrix can be functionalized with charged groups, creating a polyelectrolyte‐hydrogel with icephobic blocks and charged groups that delay ice nucleation. This system has the advantage to reduce the leaching of the charged groups since they are chemically bond to the polymeric structure. Similarly, to electrolyte hydrogel, the depression of the freezing point depends on the interaction of water with the charged groups. Differently, charged groups are not dissolved in the liquid phase but bound to the polymeric backbone. Water coordinated to the polymer ionic groups act as a lubricating layer, reducing ice adhesion.^[^ ^159^ ^]^

Finally, a further examples of gels is represented by iono‐gels, in which an ionic liquid is trapped within the polymeric matrix.^[^ ^159^ ^]^ The icephobic behavior is based on the delay of the ice nucleation due to the depression of the freezing point at the water/surface interface, so, a water droplet starts freezing at air/water interface.^[^ ^159^ ^]^ Zhuo and coworkers prepared a ionogel based on (1‐ethyl‐3‐methylimidazolium bis(trifluoromethylsulfonyl)imide, EMImTFSI) and hydrophobic polymeric matrix (poly(vinylidene fluoride‐co‐hexafluoropropylene), PVDF‐HFP). When exposed at *T* = −20 °C, a 3 µL droplet started to freeze only after 375 min, proving a successfully nucleation delay.^[^ ^217^ ^]^

#### 4.5.2 Polyelectrolyte Brushes

**Figure 7:**
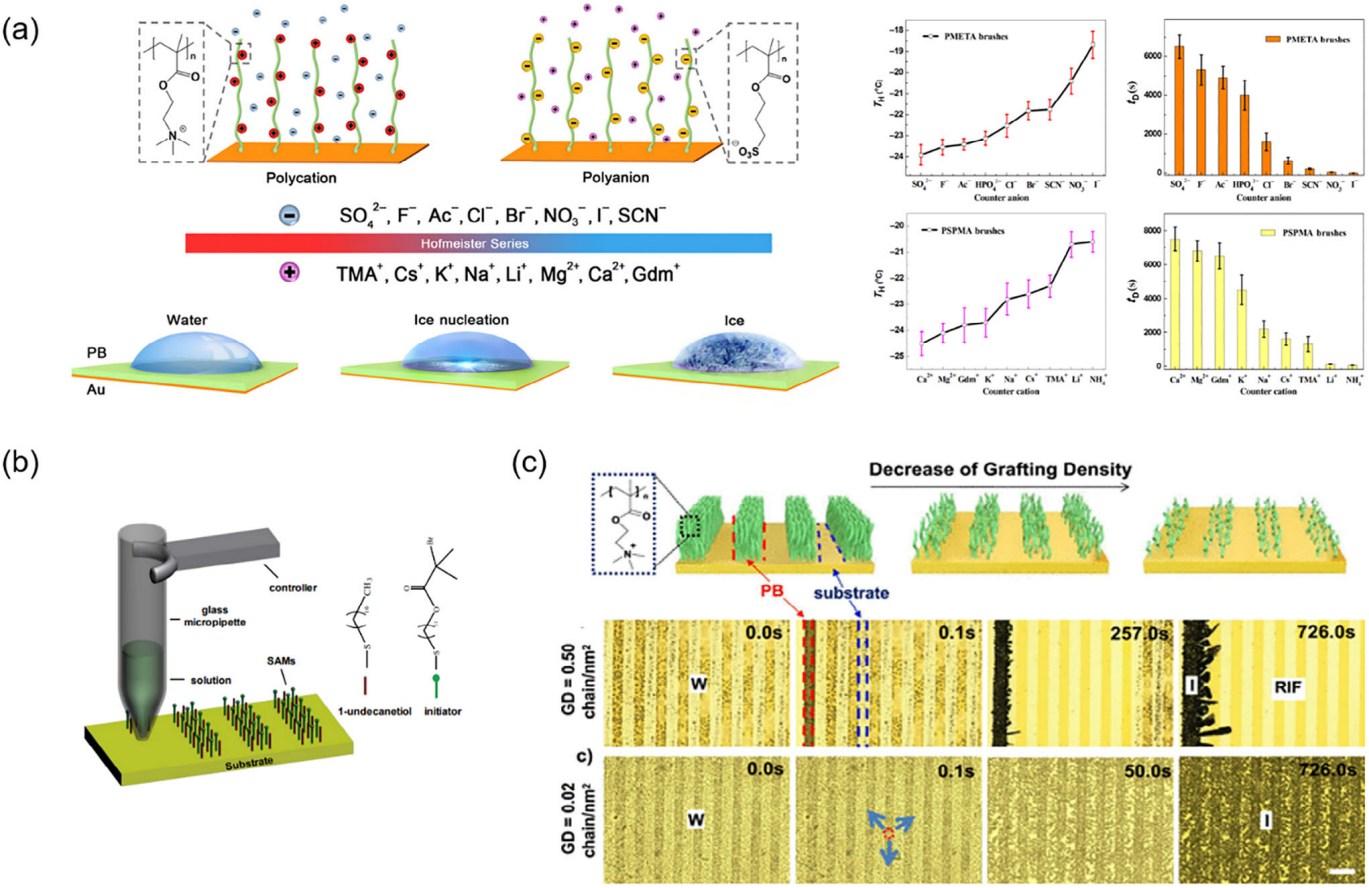
a) Examples of the structure of polyelectrolytes grafted to a surface. Ice nucleation can be tuned with counterions on brushes surfaces.Reprinted with permission.^[^ ^149^ ^]^ Copyright 2016, AAAS. b) Automatic tip to graph polyelectrolyte in specific positions on a gold substrate. The grafting solution, containing the monomer, the radical initiator, and an alkyl‐thiol, is poured over the surface by a tip. c) Optical images of ice growing on PB surfaces with patterns with different grafting density of polyelectrolyte brushes. Adapted with permission.^[^ ^150^ ^]^ Copyright 2020, American Chemical Society.

Similar to hydrogels, polyelectrolyte brushes (PE brushes) are hydrophilic. However, due to the electrostatic interaction of the charged groups, water (supercooled water droplets or humidity) coordinates to the charged groups without assuming an ice‐like configuration, allowing the depression of the freezing point. Coordinated water, because of its non‐frozen state, creates a lubricating liquid layer which lowers the ice adhesion.^[^ ^218^ ^]^ The effectiveness in lowering the freezing point depends on the grafting density of the polyelectrolyte (i.e., the density of the charged groups) and on the nature of the counter ion that follows the Hofmainster series^[^ ^149^ ^]^ (**Figure** **7**a).

Charpentier et al. patterned a steel surface by a laser ablation to create a square pillar array with micrometric size (25 µm width, 8 µm depth, 50 µm width of the pillar‐to‐pillar distance). A functionalization with methacryloxypropyltriethoxysilane, followed by a free radical polymerization of a polystyrene sulfonate polyelectrolyte, allowed to obtain an icephobic surface. The melting point was depressed up to –19.5 °C, as compared to –11 °C of the untreated steel.^[^ ^148^ ^]^ He et al. grafted anionic poly(3‐sulfopropyl methacrylate) to a gold substrate by surface initiated polymerization. The resulting freezing point was depressed to −23 °C.^[^ ^149^ ^]^ Jin et al. grafted 2‐[(methacryloyloxy)ethyl]trimethylammonium chloride in a gold substrate. Here, the precursor was poured over the substrate by a tip that can move automatically over the surface creating a regular pattern (Figure 7b). When the temperature decreased to −20 °C, the surface froze after 670 s when compared to the untreated gold substrate.^[^ ^150^ ^]^

## 5 Passive Ice Protection for Aeronautical Applications

This paragraph proposes a description of the principal passive ice protection solutions specifically adopted in the aeronautical field. A list of these treatments is reported in **Table** **5**. Coatings prepared for the aeronautical application are fabricated mainly by spray coating^[^ ^219^ ^]^ of commercial inks,^[^ ^220^ ^]^ or by etching of a metal substrate or anodization in case of aluminum.^[^ ^221^, ^222^ ^]^ Airplane components are usually special metal alloys for aeronautical applications such Aluminum Alloy 2024 (AA2024),^[^ ^28^, ^221^ ^]^ steel (Cr2Ni2WVA, aeronautic steel)^[^ ^222^ ^]^ or Titanium (Ti6Al4 V),^[^ ^100^ ^]^ that are all hydrophilic materials. Anodization of aluminum is a well‐known surface treatment which enhances the corrosion resistance properties of the aluminum alloy owing to the electrochemical oxidation of the metal to the corresponding oxide.^[^ ^223^ ^]^ This process, when combined with a chemical etching and a surface functionalization with low surface energy groups, confers icephobic properties to the alloy. Belaud et al.^[^ ^221^ ^]^ managed to prepare an icephobic aluminum surface by anodization in a sulfuric acid bath of the pretreated metallic surface (as described above), and impregnation of a commercial perfluoropolyether compound (Mecasurf). Ice adhesion strength on the as‐prepared surface was 45 kPa and the CA was 170° with a hysteresis of 4°.^[^ ^221^ ^]^ Similarly, the surface of a steel component may be oxidated to the corresponding oxide. However, due to the structure of iron oxide, this is a process that is undesired when processing steel. In this case, the electrochemical oxidation creates a rough surface, that can be turned into an icephobic component after functionalization with a FAS compound.^[^ ^222^ ^]^ Liu et al. found the as prepared component showed no ice on its surface when the untreated steel piece was completely covered by ice (same exposure time and temperature).^[^ ^222^ ^]^ Aluminum can also be transformed into an icephobic surface through the coating of polymeric ink such as fluoropolymers or commercial polyurethane paints.^[^ ^224^ ^]^

**Table 5:** Overview of commercial surface treatment for aeronautical applications.

Surface treatment	Substrate	CA [°]	Ice adhesion	Note	Refs.
Etching, anodization, perfluoropolyether impregnation	Aluminum	170	45 kPa	–	[221]
Oxydation, FAS functionalization	Steel	160	N.A.	*t* = 15 min (ice delay time)	[222]
PDMS	Aluminum	110	9 kPa		[229]
Chromium, chromium carbide, diamond like carbon, fluorine doped carbon (sputtering)	Steel	150	26 N	–	[219]
PU paint, TEOS, Glymo	Reinforced carbon fiber	83	90 kPa	Adhesion value constant for 15 cycles	[179]
PU paint, hydrophobic silica	PMMA	128	<30 mJ/m^2^		[220, 228]
PU‐Matrix, PFOA, Desmodur Z4470BA	Fiberglass	110	220 kPa	Adhesion value constant for seven cycles	[230]
Fluorinated silicone nanofilament, polyfloroether	Titanium, aluminum	160	40–65 kPa	Wettability properties stable for 16 cycles	[154]
Alumina, perfluoropolyether	Aluminum	110	23 kPa	–	[231]
PU resin, commercial silane blend (Dow corning), POSS, nano‐silica Aerosil R805	2024 aluminum alloy	116	N.A.	Ice accumulation on the coated surface is ≈48% less than the rúncoated surface at −10 °C	[232]

When UAV are considered,^[^ ^225^, ^226^, ^227^ ^]^ plastic components such as reinforced carbon fiber or polyamide are usually present. Only a few papers report a hydrophobic or an icephobic coating on plastic components. In this case, the common strategy is based on the application of a hydrophobic coating combined with low ice adhesion (see Section 4.1). A remarkable range of commercial polymeric inks are available on the market. Carreño et al. combined a commercial polyurethane paint ALEXIT with a sol‐gel coating. While polyurethane is hydrophilic, the presence of PDMS increases the water CA, and the sol‐gel coating acts as a binder layer, improving the adhesion between the coating and the carbon fiber epoxy composite.^[^ ^179^ ^]^ Similarly, Piscitelli and coworkers^[^ ^220^, ^228^ ^]^ sprayed an industrial polyurethane painting (RS 132633) and incorporated hydrophobic silica functionalized with hexamethyldisilazane over a polymethylmethacrylate substrate. In their work, the coating was icephobic after the application of 30 layers with a water CA of 128°.^[^ ^220^, ^228^ ^]^

## 6 Conclusion and Outlook

Ice protection in the aeronautical field relies on energy‐demanding systems. However, in the case of UAVs, these systems face challenges related to power constraints, weight, system integration, and high costs. As a result, passive ice protection systems have garnered increasing attention. This paper reviews a broad range of passive coatings for ice mitigation, classifying them based on their anti‐icing mechanisms: a) delaying ice nucleation while reducing ice adhesion, and b) lowering the freezing point in combination with reducing ice adhesion.

Despite significant progress in reducing ice adhesion, all proposed coatings suffer from insufficient long‐term durability, both in terms of mechanical and chemical stability. Micronanostructured SHS are unlikely to retain their ice prevention properties over time due to challenges in producing micronanopatterns smaller than ice cores and their susceptibility to corrosion and ice erosion.

The elasticity of polysiloxanes, coupled with their low surface energy, provides effective anti‐icing properties. However, their weak adhesion and low mechanical properties necessitate the development of composite materials to improve durability.

The low adhesion promoted by a lubricant layer appears to be the most promising icephobic mechanism, regardless of the chemical nature of the slippery layer (whether lubricant or “non‐frozen water”). Polyelectrolyte brushes, polyelectrolyte gels, and electrolyte gels have been shown to effectively lower the freezing point of water. However, their anti‐icing performance diminishes when the external temperature drops below the new melting point.

SLIPS exhibit interesting properties for ice protection, such as very low CA hysteresis. However, chemical stability remains a challenge, particularly due to the leaching of the lubricant. 3D LIS, characterized by a porous structure that can store lubricant, may enhance the self‐healing ability of SLIPS, thereby extending surface durability. Nevertheless, challenges persist, such as the scalability.

Recently, LLS have emerged as potential coatings to address the lubricant leaching issue of SLIPS while maintaining a low CA hysteresis. Research on LLS is still in its early stages, as evidenced by the limited number of studies published, and practical applications remain an open area of research for future development. Key questions include long‐term stability in real‐world conditions and applicability to practical rough surfaces. One possible strategy is to embed liquid‐like features into a cross‐linked polymeric network.

Embedding PCMs into a siloxane‐based coating offers another approach to ice protection. The release of latent heat due to the solidification of n‐alkane capsules near the freezing point of water can delay ice nucleation or partially melt ice on the coating, creating a slippery effect and facilitating ice removal. However, leaching of the PCM capsules remains a significant drawback of this coating system.

In addition to extending the durability of anti‐icing properties, there is an urgent need to establish standardized protocols for evaluating anti‐icing performance. While several prototype experiments have been conducted to assess the icephobicity of surfaces, no standardized characterization methods are universally recognized within the scientific community. The varying experimental conditions, such as sample dimensions, test types, and icing test conditions (temperature, humidity, undercooling, etc.), complicate the comparison of different coatings' performance. Furthermore, tests under simulated real‐world conditions, such as in a climate chamber, should be conducted.

Finally, additional challenges for applying passive systems to UAVs include scalability of the fabrication process, adaptability to nonplanar surfaces, and compatibility with the substrate, particularly concerning coating adhesion to fiber‐reinforced polymer composites. One potential solution could be combining a coating with liquid‐like properties and a cost‐effective conformal fabrication technique, such as dip coating or spray coating.

Overall, there are still several critical points to address for the practical application of passive protection systems on UAVs. In author's opinion, the main challenges are the improvement in coating adhesion to propellers and the durability of anti‐icing properties when operating in humid cold windy environments.

## Conflict of interest

The authors declare no conflict of interest.
